# Virulence gains in the *Puccinia striiformis* f. sp. *tritici PstS10* lineage correlate with expression polymorphism in a candidate Avr effector

**DOI:** 10.1038/s42003-026-10018-0

**Published:** 2026-04-13

**Authors:** Rita. Domingues Carvalho, Loizos. Savva, Julian. Rodriguez-Algaba, Andrey. Korolev, Christopher. Stephens, Anthony. Bryan, Annemarie F. Justesen, Mogens S. Hovmøller, Diane G. O. Saunders

**Affiliations:** 1 John Innes Centre, Norwich Research Park, Norwich, UK; 2 Instituto Superior de Agronomia, Lisboa, Portugal; 3 Aarhus University, Flakkebjerg, Denmark

**Keywords:** Fungal pathogenesis, Pathogens, Fungal evolution

## Abstract

Wheat yellow rust – caused by *Puccinia striiformis* f. sp. *tritici* (*Pst*) – is the most damaging wheat rust in Europe, where three distinct races (Kalmar, Amboise and Benchmark) recently became prevalent. Herein, comparative genomic analysis and pathology-based race profiling showed these races evolved from diversification in the *PstS10*/Warrior(–) lineage. Additionally, transcriptomic profiling indicated that gain of virulence in Kalmar and Amboise *Pst* isolates is seemingly associated with loss of expression of a single gene, *PST130_P495001*; Kalmar and Amboise *Pst* isolates lack *PST130_P495001* expression, whereas *Pst* isolates from Benchmark retain high expression. PST130_P495001 exhibits typical characteristics of an effector. Furthermore, examination of *PST130_P495001* across global *Pst* isolates revealed high sequence conservation, with complete loss of expression exceptionally rare (11 of 888 *Pst* datasets). *Pst* field isolates with low *PST130_P495001* expression were also found on wheat varieties with shared parentage, which only exhibited high infection levels with Kalmar-derived *Pst* isolates lacking *PST130_P495001* expression. These findings support PST130_P495001 as a candidate avirulence effector, pending further validation once the corresponding resistance gene, likely within this shared lineage, is identified. They also suggest that loss of expression may have contributed to virulence gains in the *PstS10* lineage, to overcome resistance shared by these related varieties.

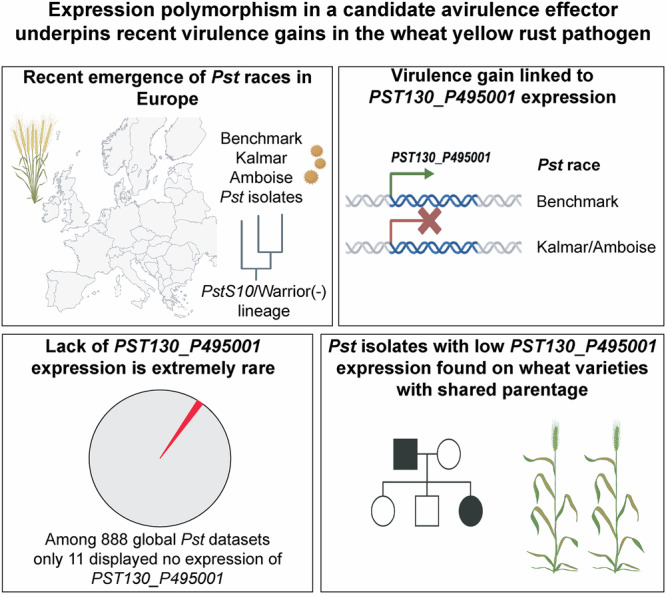

## Abstract (web summary)

Transcriptomic analyses identified loss of expression of a single candidate Avr effector gene, *PST130_P495001*, associated with a recent gain of virulence in European wheat yellow rust pathogen isolates.

## Introduction

Wheat rust fungi have been responsible for crop failures throughout history, diminishing food supplies and incurring annual global losses estimated to exceed $4 billion^1^. As with other pathogens, their parasitic lifestyle relies heavily on the secretion of effector proteins, which manipulate host cell structure and function and/or suppress host immune responses to support the pathogen’s own growth and development^2^. Among these are avirulence (Avr) effectors, encoded by *Avr* genes, which can be recognised directly or indirectly by specific host resistance (R) proteins, rendering the pathogen avirulent and restricting its growth^3^. However, this recognition process imposes strong evolutionary pressure on pathogens to modify or lose *Avr* genes, enabling them to evade immunity^4^. As a result, new virulent pathogen races (pathotypes) frequently emerge and proliferate rapidly, compromising *R*-gene mediated resistance^5^. This leads to dramatic ‘boom and bust’ cycles where effective *R*-genes are deployed across expanding geographical areas (“boom”) until virulent pathogen variants emerge (‘bust’), often resulting in catastrophic epidemics^6^. For example, in the 1970s the widespread deployment of the wheat (*Triticum aestivum*) resistance gene *Sr31* across the Americas, Europe, and Asia led to the near disappearance of the stem rust pathogen (*Puccinia graminis* f. sp. *tritici* (*Pgt*)) in the 1980s and 1990s^7^. However, when the infamous ‘Ug99’ *Pgt* race emerged in Uganda in 1998 overcoming *Sr31* resistance, this left up to 80% of the world’s wheat varieties vulnerable to infection^8^. Thus, understanding how these destructive pathogens evolve to evade host recognition is crucial for developing effective and sustainable resistance deployment strategies.

Pathogens deemed high-risk for overcoming host resistance are those with high evolutionary potential^6^. This is exemplified by the three wheat rust fungi: *Pgt*, the wheat leaf (brown) rust pathogen *Puccinia triticina* (*Pt*) and yellow (stripe) rust pathogen *Puccinia striiformis* f. sp. *tritici* (*Pst*). All three fungi exhibit large effective population sizes and rapid clonal cycles, indicative of their high evolutionary potential. Additionally, as new allele combinations (genotypes) arise they can be quickly disseminated through extensive wind-borne, long-distance spore dispersal^9^. For example, in Europe, the yellow (stripe) rust pathogen (*Pst*) is currently the most damaging of the three wheat rusts, causing recurrent crop losses of 5–10% and occasional losses reaching up to 25%^10^. However, when the multi-virulent Warrior *Pst* race (within the genetic group named *PstS7*) arrived in Europe from the near Himalayas in Asia and was detected in 2011, it spread remarkably quickly across the region. Its rapid proliferation was supported by its ability to overcome several prevailing *R*-genes in European germplasm, allowing the Warrior *Pst* race to reach high frequencies in most European countries within its first year of detection^11^. By 2014, another new *Pst* race termed *PstS10* (Warrior (–)) that had similar virulent characteristics to the *PstS7* Warrior race became dominant in Europe, further diversifying in the mid-2010’s into three distinct races: Kalmar, Amboise, and Benchmark^12^. These emergent *Pst* races were named after the three wheat varieties where virulent isolates were first detected: *cv*. Kalmar, released in 2016, *cv*. Amboise in 2017 and *cv*. Benchmark in 2012. From 2020 to 2022, these three races collectively represented 60–70% of all European *Pst* samples analysed by the Global Rust Reference Center and continue to increasingly dominate the *Pst* population landscape in the region^12^. Yet, how these races emerged and gained virulence on their namesake wheat varieties remains unknown.

The breakdown of disease resistance in crop varieties occurs when pathogens use various strategies to modify Avr effectors and evade host immunity. These strategies include sequence alterations in the *Avr* gene, cis-elements or flanking sequences, pseudogenization or complete loss of *Avr* genes, epigenetic switching of *Avr* gene expression, activation of pathogen-encoded suppressors of avirulence, among others^13,14^. However, the prevalence of comparative genomic-based approaches in the *Avr* gene discovery and analysis pipeline for the wheat rust fungi has led to a greater emphasis on *Avr* deletions and allelic variation as drivers of virulence gains. For instance, comparative genomic approaches were pivotal to the identification of the first three *Avr* genes from *Pgt* (*AvrSr50*, *AvrSr35*, and *AvrSr27*), where virulence gains in each case were associated with *Avr* gene disruption and/or allelic variation^15–17^. However, further characterisation of *Pgt AvrSr27* demonstrated that *Pgt* isolates gaining virulence to the resistance gene *Sr27* did so through a combination of *AvrSr27* deletion, copy number variation and/or expression polymorphism^17^. Similarly, expression polymorphisms were observed for *AvrLr21* from *Pt*, where evasion of recognition by the *Lr21* resistance gene in wheat was linked to altered *AvrLr21* expression levels^18^. Thus, like other pathogens, wheat rust fungi seemingly employ a variety of mechanisms to achieve virulence gains and evade host immunity.

In this study, we set out to investigate the mechanisms underpinning the recent virulence gains that facilitated emergence of the Kalmar, Amboise, and Benchmark *Pst* races. Comparative genomic analyses and pathology-based race profiling of representative *Pst* isolates from these races confirmed they likely emerged through diversification in the Warrior(-) race within the *PstS10* genetic group in Europe. Furthermore, we found that the recent gain of virulence in the Kalmar and Amboise *Pst* isolates was seemingly associated with expression polymorphism in a single gene, *PST130_P495001*. This gene encodes a protein exhibiting all the typical characteristics of an effector, including a functional signal peptide at the N-terminus. Further analysis of *PST130_P495001* across 273 global *Pst* isolates revealed that its sequence is unusually highly conserved, with complete loss of *PST130_P495001* expression only observed in 11 additional *Pst* datasets out of 888 analysed. Furthermore, examination of *PST130_P495001* expression in *Pst*-infected field samples indicated that isolates with low expression were mainly found on wheat varieties with a shared parental lineage. Notably, these varieties only exhibited high levels of infection with Kalmar-derived *Pst* isolates lacking *PST130_P495001* expression, but not with the Benchmark *Pst* isolates, which retained normal expression levels. In addition, artificial suppression of *PST130_P495001* expression was shown to substantially enhance *Pst* infection of the Kalmar variety for a *Pst* isolate usually avirulent on Kalmar. These findings strongly support PST130_P495001 as a compelling candidate for an Avr protein, with the possibility that these varieties carry a corresponding resistance gene, inherited through their shared parentage.

## Results

### Diversification in the *PstS10* race group led to a recent gain of *Pst* virulence in Europe

**Fig. 1:**
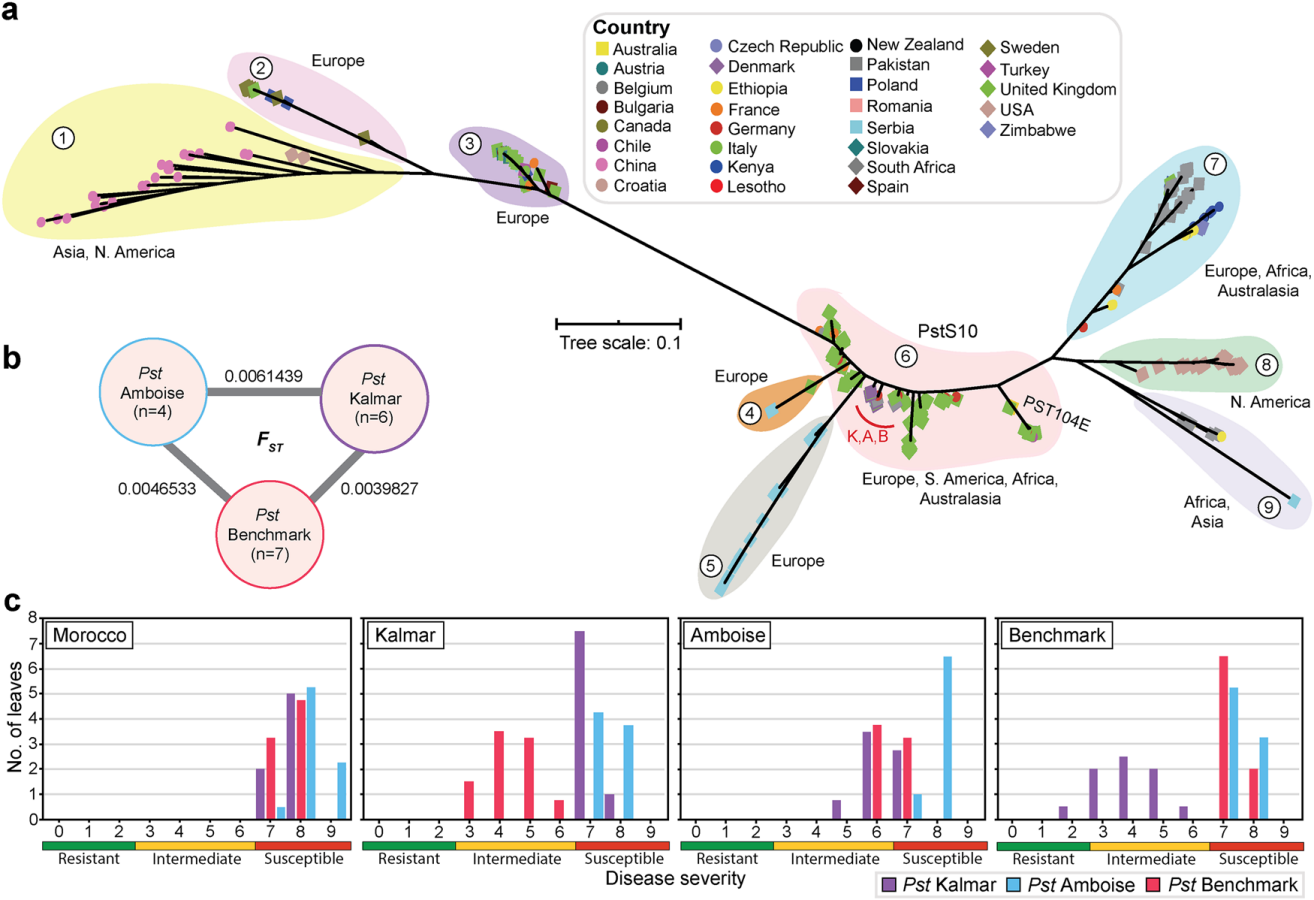
Diversification in the *PstS10* race group led to emergence of the Kalmar, Amboise and Benchmark *Pst* isolates. **a** Phylogenetic analysis illustrated that *Pst* isolates collected on the Kalmar, Amboise and Benchmark wheat varieties (‘K, A, B’) cluster within a single diverse clade (Clade 6) that also contains *Pst* isolates from the *PstS10* race group and the genome reference isolate (Pst104E137A-). Phylogenetic relationships were inferred using an approximate maximum-likelihood model based on 433 genomic or transcriptomic *Pst* datasets, identifying nine distinct clades (coloured backgrounds). Scale bar indicates the mean number of nucleotide substitutions per site. **b** Pairwise analysis of the Benchmark, Kalmar, and Amboise *Pst* isolates confirmed a close genetic relationship, with Weir and Cockerham fixation index (*F*_*ST*_) values ranging from 0.0039827 to 0.0061439. Genome-wide *F*_*ST*_ values were determined for each *Pst* isolate from Kalmar (6 isolates), Amboise (4 isolates) and Benchmark (7 isolates) to estimate the level of genetic differentiation between the three *Pst* isolate groups. **c** *Pst* isolates collected on the Benchmark, Kalmar and Amboise wheat varieties varied in their virulence profile on Benchmark, Kalmar and Amboise. Each *Pst* isolate was inoculated onto the three wheat varieties at seedling stage and disease severity assessed approximately 16 days after inoculation. Infection types were classified as resistant (green line: 0, 1, 2), intermediate (yellow line: 3, 4, 5, 6) and susceptible (red line: 7, 8, 9). Infection types on the ‘Morocco’ wheat variety were included as a universally susceptible control.

To investigate the genetic relationships among the recently emergent *Pst* isolates in Europe, RNA-seq analysis was performed on *Pst* isolates originally collected on wheat varieties Kalmar (6 isolates), Amboise (4 isolates) and Benchmark (7 isolates). To also compare these *Pst* isolates to those prevalent globally, publicly available genomic and transcriptomic data from an additional 416 *Pst* isolates collected across 29 countries were included (Supplementary Data 1). All datasets were aligned to the *Pst* reference genome (*Pst* isolate 104E137A-^19^), with 54.6% (SD 5.7%) of reads aligned for transcriptomic datasets and 67.2% (SD 8.3%) for genomic datasets (Supplementary Data 2). Considering the *Pst* isolates collected on the Kalmar, Benchmark and Amboise wheat varieties, an average of 65.2% (SD 2.0%) aligned. Phylogenetic analysis grouped the 433 *Pst* isolates into 9 distinct clades, with all European *Pst* isolates distributed across 6 clades (Fig. 1a). These included clades 2–5, which comprised of *Pst* isolates exclusively collected in Europe since 2011, as well as clade 6, the largest group containing 191 *Pst* isolates collected across Europe, South America, Africa and Australasia since 1978. Clade 7, which included 68 *Pst* isolates, spanned Europe, Africa and Australasia and dated from 2014 onwards (Supplementary Data 1). Notably, Clade 6 contained *Pst* isolates belonging to the *PstS10* race group, which has become dominant in many European countries since 2012^12^. All recent *Pst* isolates collected on the Kalmar, Benchmark and Amboise wheat varieties were found within a single clade, Clade 6, suggesting these races likely emerged through diversification within this lineage. Assessment of the level of genetic differentiation between recently emergent *Pst* isolates further supports a close genetic relationship, with *F*_*ST*_ values ranging from 0.0039827 to 0.0061439 (Fig. 1b).

To assess the virulence profiles of *Pst* isolates recently identified on the wheat varieties Kalmar, Amboise and Benchmark, representative *Pst* isolates from each variety were collected for analysis. Following multiplication, urediniospores for these *Pst* isolates were independently used to inoculate a standard set of wheat differential lines, which also included the Kalmar, Amboise and Benchmark varieties. Disease severity was assessed approximately 16 days post-inoculation (dpi) in seedling assays. All *Pst* isolates displayed a virulence profile consistent with *PstS10* race characteristics, being virulent on wheat lines carrying *Yr1*, *Yr2*, *Yr3*, *Yr4*, *Yr6*, *Yr7*, *Yr9*, *Yr17*, *Yr25*, *Yr32*, *YrSP* and *YrAvS*, and avirulent on wheat lines carrying *Yr5*, *Yr8*, *Yr10*, *Yr15*, *Yr24* and *Yr27*^12^ (Supplementary Table 1). However, additional variation was observed: *Pst* isolates from Kalmar were partially virulent on Amboise, while *Pst* isolates from Amboise showed virulence on both Kalmar and Benchmark. *Pst* isolates from Benchmark were avirulent on Kalmar but virulent on Benchmark and partially virulent on Amboise (Fig. 1c, Supplementary Fig. 1 and Supplementary Data 3). Overall, this analysis indicates that the emergence of these closely related *Pst* isolates likely resulted from minor genetic and phenotypic diversification within the *PstS10* race group.

### Gain of virulence in the *PstS10* race group is linked to expression polymorphism in *PST130_P495001*

**Fig. 2:**
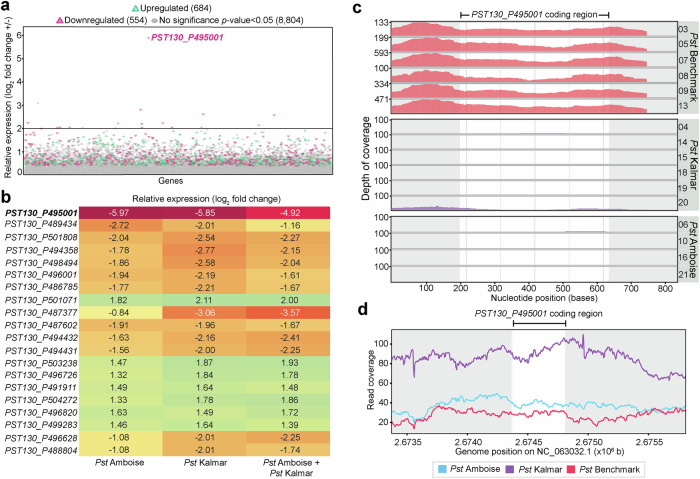
*Pst* isolates from Kalmar and Amboise display loss of expression in *PST130_P495001* that is not attributed to gene deletion. **a** Pairwise comparisons of transcript abundance between *Pst* isolates from Kalmar (6 isolates) against *Pst* isolates from Benchmark (7 isolates) identified *PST130_P495001* as a differentially expressed gene. **b** Further assessment of transcript abundance between *Pst* isolates from Kalmar (6 isolates) and/or Amboise (4 isolates) against *Pst* isolates from Benchmark (7 isolates) revealed distinct differential expression patterns, with *PST130_P495001* displaying the most significant fold change (*p-*value < 0.001) in all comparisons. Transcript abundance was assessed for each of the 17 *Pst* isolates using pseudoalignments to the *Pst* reference genome available in the Ensembl BioMart database (isolate PST-130^20,21^). **c** Six Kalmar *Pst* isolates and all four *Pst* isolates from Amboise displayed a complete lack of *PST130_P495001* expression, which remains consistently and highly expressed in all *Pst* isolates from Benchmark. Coloured blocks, depth of coverage; white boxes, coding region of *PST130_P495001*. **d** Assessment of read coverage following alignment of the *PST130_P495001* gene locus (contig NC_063032.1) indicates complete gene coverage in the Kalmar, Amboise and Benchmark *Pst* isolates. Long-read genome sequencing was performed on three representative *Pst* isolates (one each from the wheat varieties Kalmar, Amboise and Benchmark) and aligned to the *Pst* reference genome (isolate 134E16A + 17 + 33 + ^19^). White box, coding region of *PST130_P495001*.

To investigate genetic signatures that could be associated with the differences in virulence specificities among *Pst* isolates collected from Kalmar, Benchmark and Amboise, we analysed gene sequence and expression polymorphisms across the three *Pst* isolate groups. Synthetic gene sets were generated for each *Pst* isolate, incorporating all single-nucleotide polymorphisms (SNPs) identified through alignment to the *Pst* reference genome (isolate 104E137A-^19^). Pairwise sequence comparisons of the 30,249 gene models among the Kalmar- (6 isolates), Amboise- (4 isolates) and Benchmark- (7 isolates) race groups revealed no specific gene isoforms that could explain the differences in virulence profiles (Supplementary Fig. 2a and Supplementary Data 4). Next, gene expression profiles were analysed by assessing transcript abundance for each of the 17 *Pst* isolates using pseudo-alignment to the *Pst* reference genome available in the Ensembl BioMart database (isolate PST-130^20,21^). Pairwise comparisons of transcript abundance between *Pst* isolates from Kalmar (6 isolates) or Amboise (4 isolates), against *Pst* isolates from Benchmark (7 isolates) revealed distinct differential expression patterns (Supplementary Data 5). In Kalmar *Pst* isolates compared to Benchmark, 684 genes were up-regulated, including three with a fold change greater than 2, while 554 genes were down-regulated, with 12 showing a fold change less than −2 (Fig. 2a). In Amboise *Pst* isolates compared to Benchmark, none of the up-regulated genes (4913 total) showed a fold change greater than 2, while three of the 4697 down-regulated genes had a fold change less than −2 (Supplementary Fig. 2b). Among these, one gene (*PST130_P495001*) showed the most significant fold changes: −5.8508 (*p*-value 4.0022e^−26^) and −5.9730 (*p*-value 6.1884e^−52^) when comparing *Pst* isolates from Kalmar and Amboise to those from Benchmark, respectively (Fig. 2b). Further examination of *PST130_P495001* transcript abundance revealed a complete lack of expression in all but one of the six Kalmar *Pst* isolates and in all four *Pst* isolates from Amboise, whereas *Pst* isolates from Benchmark retained consistently high levels of expression (Fig. 2c).

To determine if the loss of *PST130_P495001* expression in *Pst* isolates identified on Kalmar and Amboise could be attributed to deletion of the gene locus, we performed genome sequencing on three representative *Pst* isolates: one each from the wheat varieties Kalmar, Amboise and Benchmark. Long-read sequencing data were aligned to the *Pst* reference genome (isolate 134E16A + 17 + 33 + ^19^) and analysis of the sequence alignments confirmed the presence of the complete *PST130_P495001* locus in all three *Pst* isolates (Fig. 2d). To investigate potential sequence changes in the surrounding regulatory regions, we aligned sequences 2 Kb upstream and downstream of the *PST130_P495001* coding sequence (CDS). The alignments showed complete sequence conservation within the flanking regions across the three *Pst* isolates (Supplementary Fig. 3). Complete sequence conservation was further confirmed in alignment to the alternative *Pst* reference genome (isolate 134E16A + 17 + 33 + ^19^) (Supplementary Fig. 3). Finally, we searched for any repeat elements that could be influencing gene expression within 20 Kb flanking regions of the *PST130_P495001* CDS and for comparison within 20 Kb regions surrounding four housekeeping genes (*Elongation Factor 1*, *Beta-tubulin*, *Alpha-tubulin* and *Actin*) in the *Pst* reference genome (isolate 134E16A + 17 + 33 + ^19^). This analysis identified seven simple repeat elements within the *PST130_P495001* locus that were similar to those located near one or more housekeeping genes that are invariably expressed (Supplementary Fig. 4). These findings suggest that the loss of *PST130_P495001* expression in *Pst* isolates from Kalmar and Amboise is unlikely to be caused by deletion or sequence diversification in the *PST130_P495001* locus.

### PST130_P495001 is a secretory protein that localises to the cytoplasm and nucleus *in planta*

**Fig. 3:**
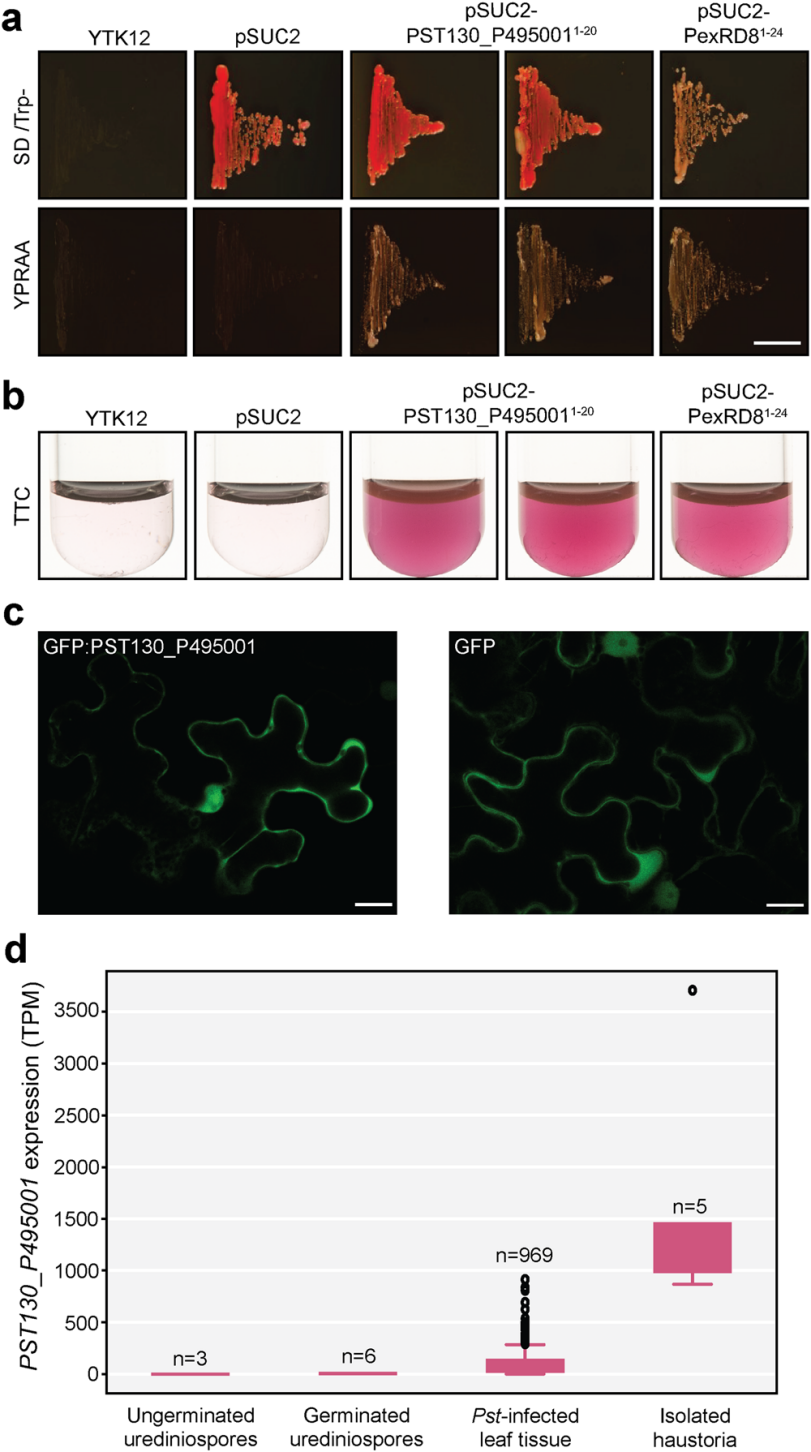
PST130_P495001 has a functional signal peptide in the N-terminus, localises to the cytoplasm and nucleus and is highly expressed in isolated *Pst* haustoria. **a** The yeast YTK12 strain harbouring pSUC2-PST130_P495001^1–20^ grew well on SD/Trp- medium, indicating successful transformation and on restrictive media with raffinose as the sole carbon source (YPRAA media) confirming PST130_ P495001^1–20^ secretory function. The invertase secretion-deficient yeast strain YTK12 was transformed with pSUC2, pSUC2-PST130_P495001^1–20^ or the pSUC2-PexRD8^1–24^ positive control^23^. Scale bar represents 10 mm. **b** In enzymatic activity assays only YTK12 strains carrying pSUC2-PST130_P495001^1–20^ or pSUC2-PexRD8^1–24^ successfully reduced triphenyltetrazolium chloride (TTC) to the red insoluble triphenylformazan (TF), confirming the secretion of invertase. **c** PST130_P495001 localised predominantly to the cytoplasm and nucleus in a distribution indistinguishable to the GFP control. GFP:PST130_P495001 and GFP were independently transiently expressed in *N. benthamiana* leaf cells and images captured 3 days post-infiltration. Scale bars represent 20 μm. **d** *PST130_P495001* is highly expressed in isolated *Pst* haustoria when compared to other infection structures, such as ungerminated urediniospores, germinated urediniospores and *Pst*-infected wheat leaf tissue. Expression analysis was conducted using 992 publicly available RNA-seq datasets^25–27^.

To determine whether *PST130_P495001* encodes a *Pst* protein with potential *in planta* function, we examined the corresponding amino acid sequence for characteristics typical of secreted effector proteins. The first 17 amino acids of PST130_P495001 were predicted to encode a signal peptide (SP)^22^ (Supplementary Table 2), which was tested for secretory function. The invertase secretion-deficient yeast strain YTK12 was transformed with pSUC2, pSUC2-PST130_P495001^1–20^ or the pSUC2-PexRD8^1–24^ positive control^23^. After 2 days of incubation, YTK12 strains were assessed for growth. All transformed strains grew well on SD/Trp- medium, confirming successful transformation (Fig. 3a). However, on restrictive media with raffinose as the sole carbon source (YPRAA media), YTK12 strains carrying pSUC2-PST130_P495001^1–20^ or pSUC2-PexRD8^1–24^ exhibited robust growth, whereas YTK12 strains with the empty pSUC2 vector or no vector lacked growth (Fig. 3a). These results were corroborated by enzymatic activity assays. Only YTK12 strains carrying pSUC2-PST130_P495001^1–20^ or pSUC2-PexRD8^1–24^ successfully reduced triphenyltetrazolium chloride (TTC) to the red insoluble triphenylformazan (TF) indicating invertase secretion (Fig. 3b).

Further analysis of *PST130_P495001* revealed it encoded a protein with unknown function which is a typical characteristic of effector proteins and was also predicted by machine learning to be a dual localised (apoplastic/cytoplasmic) effector protein^24^ (Supplementary Table 2). To investigate the spatial distribution of PST130_P495001, we fused GFP to the mature form of PST130_P495001 and expressed the fusion protein (GFP:PST130_P495001) heterologously in *Nicotiana benthamiana*. Assessment of the intracellular localisation by confocal microscopy, indicated that GFP:PST130_P495001 accumulated largely in the cytoplasm and nucleus, with a distribution indistinguishable from the GFP control (Fig. 3c). Expression analysis using publicly available RNA-seq datasets^25,26^ also showed that *PST130_P495001* is highly expressed in isolated *Pst* haustoria when compared to other infection structures, such as ungerminated urediniospores, germinated urediniospores and *Pst*-infected wheat leaf tissue (Fig. 3d). Overall, these findings confirm the presence of a functional signal peptide in the N-terminus of PST130_P495001 and demonstrate that PST130_P495001 possesses several features typical of an effector protein.

### *PST130_P495001* is highly conserved within the global *Pst* population, with loss of expression rare

**Fig. 4:**
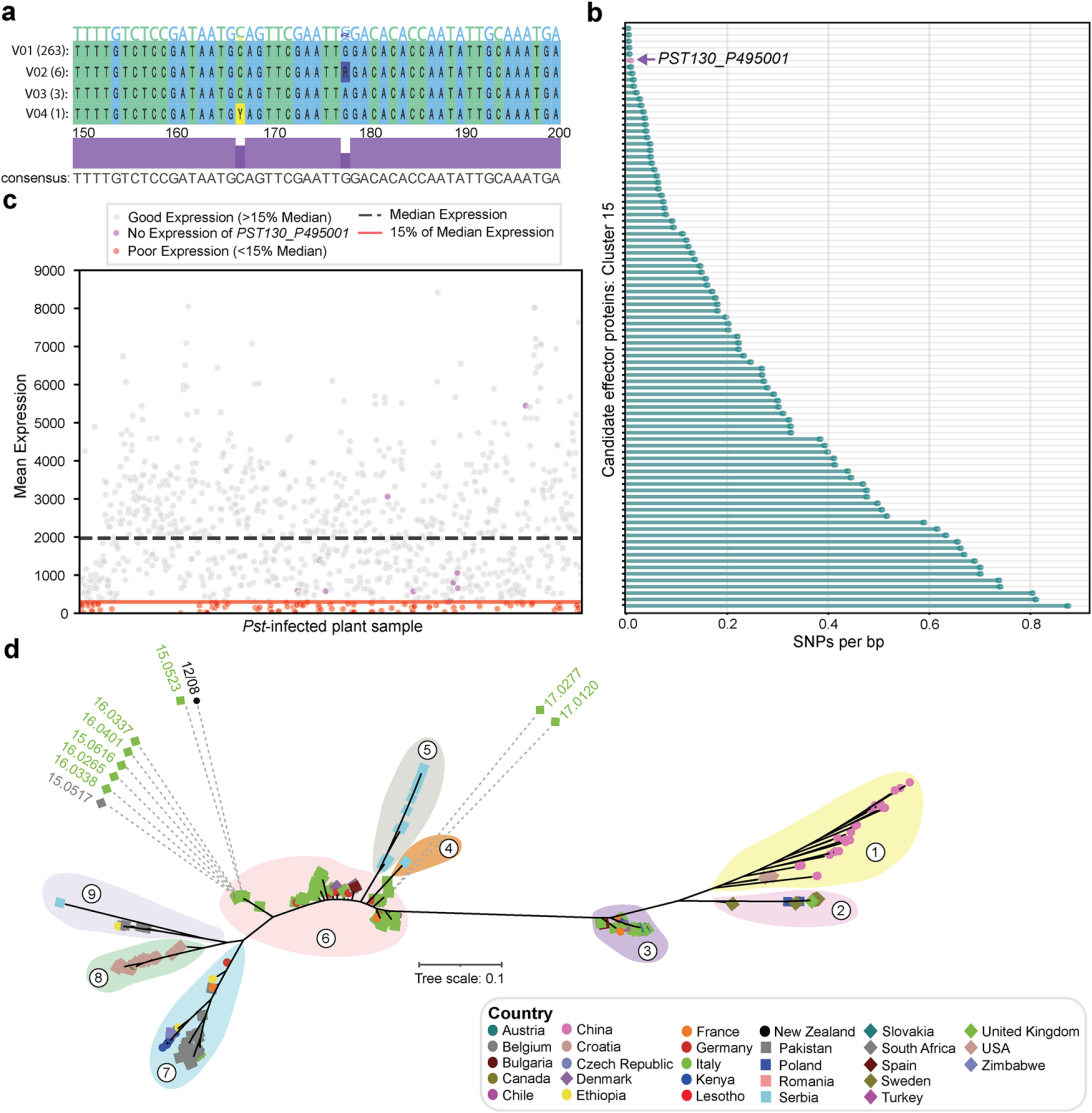
*PST130_P495001* encodes a highly conserved putative *Pst* effector protein, with loss of expression rare across global *Pst* isolates. **a** Four sequence variant combinations of *PST130_P495001* were identified across 273 global *Pst* isolates, with variant 1 (V01) being the most prevalent (263 out of 273 *Pst* isolates). *PST130_P495001* sequences were extracted following alignment of each *Pst* dataset to the *Pst* reference genome (isolate 104E137A-^19^). Parentheses, number of *Pst* isolates displaying the given *PST130_P495001* variant combination. **b** Among 91 genes previously grouped with *PST130_P495001*, based on expression profile similarity (referred to as cluster 15^19^), *PST130_P495001* ranked in the top 10% of the most sequence conserved genes in this subset. Sequence variation was analysed for 91 of the 156 genes in this gene cluster that were located in available datasets for 273 global *Pst* isolates. The dashed line denotes the 10th percentile threshold of conservation. **c** Among 888 global *Pst* datasets only 10 collected on *T. aestivum* (and one on *T. durum* see Supplementary Fig. 6) displayed no detectable *PST130_P495001* expression. Transcript counts for each *Pst* isolate were extracted from publicly available *Pst* gene expression data^27^. Grey dots, *Pst* isolates with good expression (>15% median); orange dots, *Pst* isolates with low expression (<15% median); purple dots, *Pst* isolates with no detectable *PST130_P495001* expression; dashed line, median expression level; solid orange line, 15% median expression. **d** Phylogenetic analysis indicated that *Pst* isolates with no detectable *PST130_P495001* expression, were distributed across multiple branches within clade 6 in the phylogeny. Phylogenetic relationships were inferred using an approximate maximum-likelihood model based on 437 genomic or transcriptomic *Pst* datasets that included 10 *Pst* datasets lacking *PST130_P495001* expression. Nine distinct clades are indicated (coloured backgrounds). Scale bar indicates the mean number of nucleotide substitutions per site.

To investigate the conservation of *PST130_P495001* across *Pst* isolates, we analysed the *PST130_P495001* locus using data from 413 global *Pst* isolates spanning 29 countries. Each dataset was aligned to the *Pst* reference genome (isolate 104E137A-^19^), and sequences corresponding to the *PST130_P495001* gene were successfully extracted for 273 of the datasets. Across these *Pst* isolates, we observed an average of 0.0024 (SD. 0.0004) SNPs per base in *PST130_P495001*. For comparison, the average SNPs per base rates were 0.0320 (S.D. 0.0005), 0.0073 (S.D. 0.0009) and 0.0422 (S.D. 0.0004) for three highly conserved genes: *Actin* (*PST104_9243*), *Beta-tubulin* (*PST104_20937*) and *Elongation Factor 1* (*PST104_11781*), respectively (Supplementary Data 6). Only 10 of the 273 *Pst* isolates (3.42%) exhibited sequence polymorphisms in *PST130_P495001* that could lead to non-synonymous substitutions. This included nine *Pst* isolates from China with a G60R substitution, where six *Pst* isolates were homokaryotic and three heterokaryotic for the substitution, and one *Pst* isolate from Denmark with a heterokaryotic A56V substitution (Fig. 4a and Supplementary Fig. 5a, b). Further analysis of the *PST130_P495001* sequence within the phased *Pst* reference genome assembly (*Pst* isolate 104E137A-^19^) confirmed its presence in both haplotypes, with at least one *PST130_P495001* homologue identified in all 19 *Pst* genome assemblies examined (Supplementary Fig. 5c and Table 3). To determine whether similar sequence conservation levels were observed for other candidate secreted effector proteins (CSEPs), we analysed the sequences of 156 genes previously grouped with *PST130_P495001* based on similarities in their expression profiles (referred to as cluster 15^19^). Sequence similarity searches identified homologues for 91 of these genes in the *Pst* reference genome (isolate PST-130^20^). Assessment of the SNPs per base rates across the same 273 global *Pst* isolates, revealed that *PST130_P495001* ranked in the top 10% of the most conserved CSEPs in this cluster (Fig. 4b), further highlighting PST130_P495001 as a highly conserved CSEP in *Pst*.

Given that *PST130_P495001* is highly conserved among *Pst* isolates, we sought to investigate the frequency of loss of expression as a potential alternative mechanism for evading putative effector recognition. To this end, we analysed *PST130_P495001* expression across available RNA-seq datasets from 992 global *Pst* isolates^27^ (Supplementary Data 7). First, to identify datasets with low coverage that could skew the results we examined the expression of four housekeeping genes: *Actin* (*PST130_09225*), *Alpha-Tubulin* (*PST130_10193*), *Beta-Tubulin* (*PST130_06515*) and *Elongation Factor 1* (*PST130_14067*). Datasets with transcript counts below 15% of the median for these housekeeping genes were excluded from further analysis, resulting in 888 *Pst* datasets (89.5%) for analysis (Supplementary Data 7). Among these, ten *Pst* datasets from bread wheat (*Triticum aestivum*) and one from durum wheat (*Triticum durum*) showed no detectable *PST130_P495001* expression (Fig. 4c). We noted the earliest *Pst* isolate lacking *PST130_P495001* expression was collected from the bread wheat variety Claire in New Zealand in 2012 (12/08) (Supplementary Data 7). To examine the genetic similarity of the *Pst* isolates lacking *PST130_P495001* expression, we aligned each dataset to the *Pst* reference genome (isolate 104E137A-^19^) and performed phylogenetic analysis. This analysis also included an additional set of 423 *Pst* isolates to position the isolates in a global context (Supplementary Data 1). Phylogenetic analysis revealed that *Pst* isolates lacking *PST130_P495001* expression were distributed across multiple branches within the highly diverse clade 6 that also contains the *PstS10* race group (Fig. 4d and Supplementary Fig. 6). This suggests that loss of *PST130_P495001* expression could have arisen independently in several *Pst* lineages.

### Wheat varieties infected by *Pst* isolates with low *PST130_P495001* expression have a shared parental lineage

**Fig. 5:**
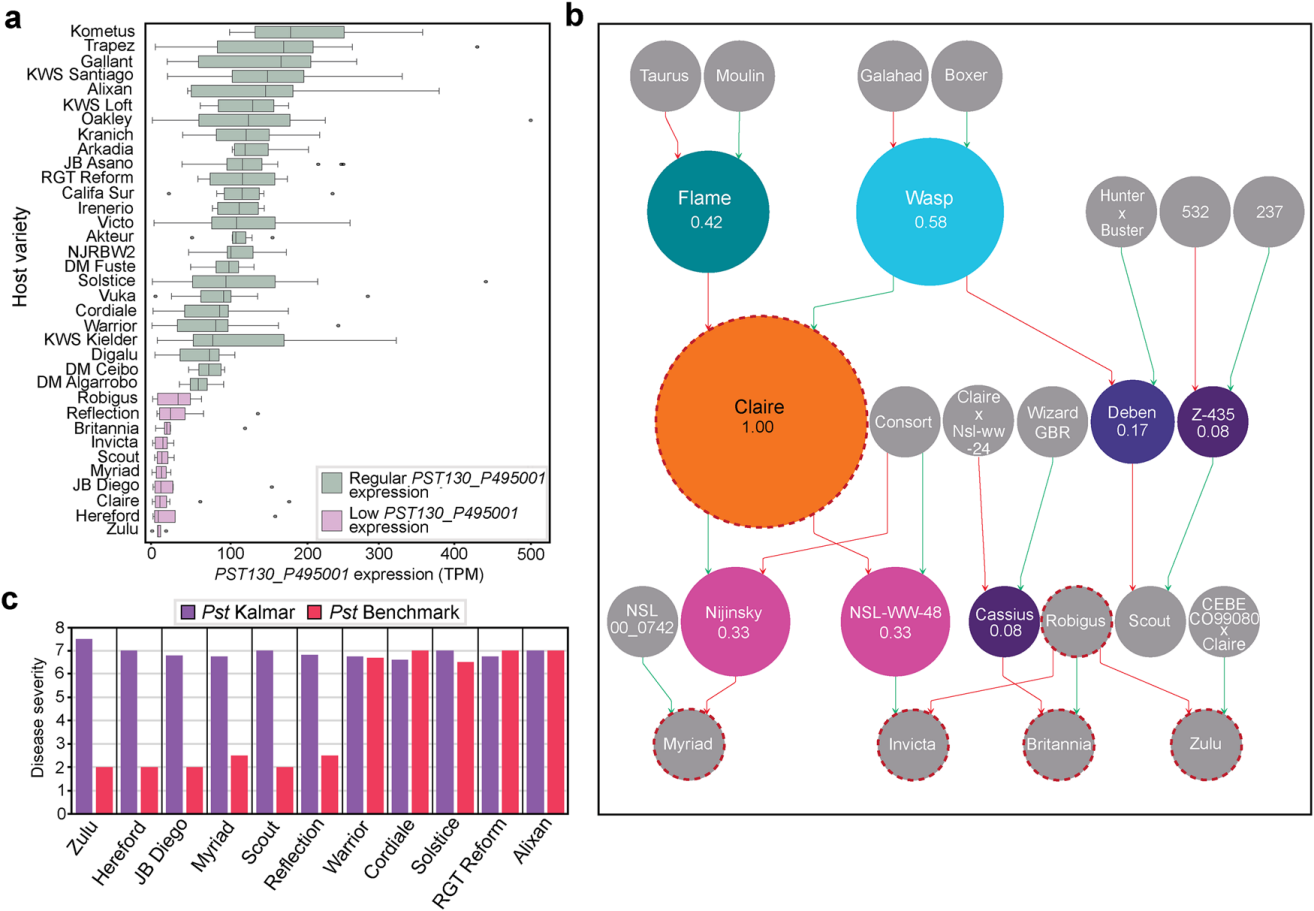
*Pst* isolates with low *PST130_P495001* expression were predominantly found on wheat varieties with shared parentage. **a** A total of 10 wheat varieties were infected with *Pst* isolates with a significantly lower median expression of *PST130_P495001* compared to the overall population median (*padj* < 0.4 and abs(log_2_(Fold Change) > 0.75). Transcripts per million (TPM) counts for *PST130_P495001* were assessed in publicly available RNA-seq data for *Pst*-infected field samples collected across 35 wheat varieties, where at least four *Pst*-infected samples were available^27^. Bars represent median values, boxes signify the upper (Q3) and lower (Q1) quartiles, and whiskers are located at 1.5 the interquartile range. **b** Of the nine wheat varieties infected by *Pst* isolates with low expression and known parentage, three wheat varieties – Claire, Wasp and Robigus – were frequently found in the pedigrees. The network displays the first- and second-generation ancestors of Claire, Wasp and Robigus, with red arrows indicating the masculine parent and green arrows the feminine parental contributions. Node sizes, numerical values; colours, betweenness in pedigrees (Claire: 1.00, Wasp: 0.58, Flame: 0.42, Nijinsky: 0.33, NSL-WW-48: 0.33, Deben: 0.17, Z-435: 0.08, Cassius: 0.08); dashed red circle, varieties infected by *Pst* isolates with low *PST130_P495001* expression. **c** Disease severity for wheat varieties linked to *Pst* isolates displaying low *PST130_P495001* expression (Zulu, Hereford, JB Diego, Myraid, Scout and Reflection) varied depending on *PST130_P495001* expression of the infecting *Pst* isolate. One *Pst* isolate with low *PST130_P495001* expression (*Pst* Kalmar) and one displaying regular *PST130_P495001* expression (*Pst* Benchmark) were used to inoculate 11 wheat varieties at seedling stage and disease severity assessed approximately 16 days after inoculation. Infection types were classified as resistant (0, 1, 2), intermediate (3, 4, 5, 6) and susceptible (7, 8).

Loss of *PST130_P495001* expression could be reflective of the gene encoding a typical avirulence protein, where the absence of expression could facilitate *Pst* infection in wheat varieties containing the corresponding *R* gene. To explore whether wheat varieties infected by *Pst* isolates with low *PST130_P495001* expression inherited a corresponding *R* gene through shared heritage, we examined their parentage. We obtained available RNA-seq data for *Pst*-infected field samples collected across 35 wheat varieties, where at least four *Pst*-infected samples were available^27^. The median level of *PST130_P495001* expression was assessed, revealing 10 wheat varieties infected with *Pst* isolates with a significantly lower median expression of *PST130_P495001* compared to the overall population median (*padj* < 0.4 and abs(log_2_(Fold Change) > 0.75) (Fig. 5a and Supplementary Data 8). To trace the parental lineages of each variety, we used a previously published wheat pedigree dataset encompassing pedigree details for >1,800 wheat accessions^28^, which filtered our wheat varieties to 20 with complete pedigree data (nine infected with *Pst* isolates with “low *PST130_P495001* expression” and 11 other varieties). Analysis of the first- and second-generation parental lineages identified three wheat varieties – Claire, Wasp and Robigus – as frequently present in the pedigrees of varieties infected with *Pst* isolates exhibiting ‘low *PST130_P495001* expression’ (4 out of 9; Fig. 5b). These varieties were largely absent from the pedigree of the other varieties (1 in 11; (Supplementary Table 4)). These findings suggest that wheat varieties infected by *Pst* isolates with ‘low *PST130_P495001* expression’ may have inherited a corresponding resistance locus through shared ancestry.

To further explore the potential link between *PST130_P495001* expression and *Pst* infection severity, we selected 11 wheat varieties for further analysis. *Pst* urediniospores from *Pst* isolates originally collected on Kalmar or Benchmark were independently used to inoculate each wheat variety in a seedling infection assay, with infection severity assessed 16 dpi (Supplementary Table 5). The results showed that wheat varieties typically infected by *Pst* isolates with regular levels of *PST130_P495001* expression were all highly susceptible to both *Pst* isolates (Fig. 5b). In contrast, wheat varieties associated with *Pst* isolates showing low *PST130_P495001* expression exhibited high levels of infection only when infected with the *Pst* isolate from Kalmar that lacks *PST130_P495001* expression. When inoculated with the Benchmark-derived *Pst* isolate that has regular *PST130_P495001* expression, these 6 wheat varieties were classified as resistant (Fig. 5c). This analysis further indicates that reduced *PST130_P495001* expression in the recently emerged *Pst* isolates from Kalmar may have facilitated infection of wheat varieties that predominantly share a common parental lineage.

### PST130_P495001 exhibits features typical of an avirulence protein, and suppression of its expression can induce *Pst* virulence on Kalmar

**Fig. 6:**
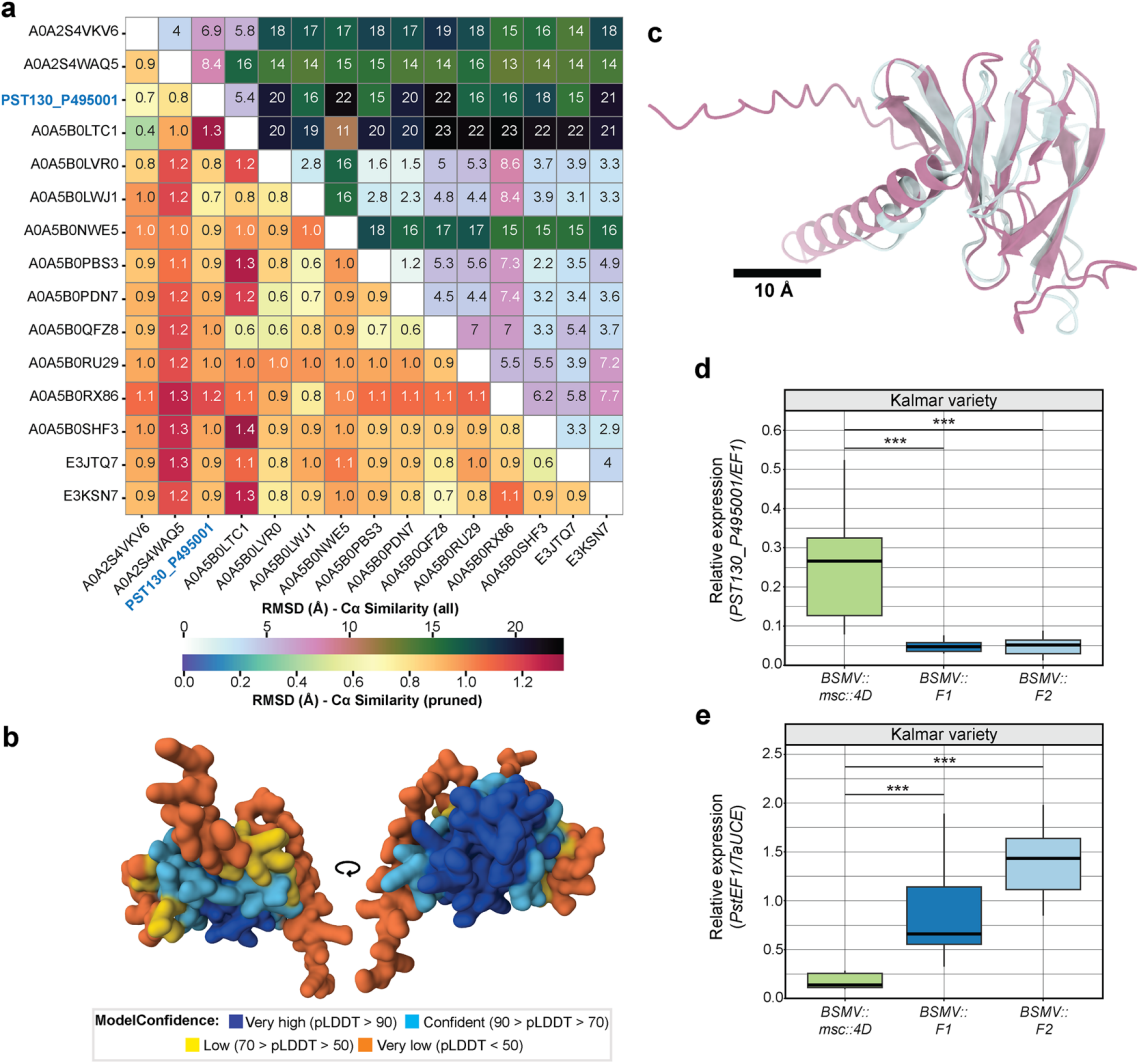
PST130_P495001 shares similar characteristics to predicted effector proteins from other *Puccinia* species, and its suppression can lead to a gain in *Pst* virulence on Kalmar. **a** Root-mean-squared-deviation (RMSD) matrix illustrating the close structural homology within the superimposed backbone-C (Cα) chain of PST130_P495001 and twelve predicted effector proteins from *Puccinia graminis* f. sp. *tritici* (*Pgt*) and two from *Puccinia striiformis* f. sp. *hordei* that clustered using Foldseek and MMseqs2. **b** The predicted 3D structure of PST130_P495001 contains two high-confidence anti-parallel β-sheets and a C-terminal α-helix, which is consistent with several characterised fungal Avr effectors. **c** Superimposed PST130_P495001 (pink; UniProt ID: A0A2S4WAX4) and *Pgt* AvrSr50 (pale blue; PDB ID: 7mqq) protein structures reveal matching secondary structural characteristics, with an RMSD of 1.2 Å and 9.2 Å for the position of Cα in pruned and all atom pairs. **d**, **e** Suppression of *PST130_P495001* expression substantially enhanced *Pst* infection of the Kalmar variety for an avirulent *Pst* isolate. Host-induced gene silencing (HIGS) of *PST130_P495001* was conducted using two independent fragments of the *PST130_P495001* gene (F1 and F2) and a Benchmark-derived *Pst* isolate (DK229_19) during infection of the Kalmar variety. *PST130_P495001* silencing and fungal biomass were assessed using RT-qPCR at 4 days-post infection (dpi) and 11-15 days post-viral inoculation (dpvi) by comparing *PST130_P495001* to *PstEF1* expression (**d**) or *PstEF1* expression to the *TaUCE* wheat reference gene (**e**). Two to four samples each were evaluated for *BSMV::F1*, *BSMV::F2*, and *BSMV::msc4D*. Asterisks denote statistically significant differences between each pair of conditions (***: *p* < 0.001; two-tailed *t*-test). Bar represents median value, box signifies the upper (Q3) and lower (Q1) quartiles, and whiskers are located at 1.5 times the interquartile range.

To assess the level of conservation for PST130_P495001 among other fungal species, we examined sequence similarity and structural homology. A sequence similarity search was performed for PST130_P495001 against the NCBI non-redundant protein sequence database. This analysis indicated that PST130_P495001 is only present in *Pst and Puccinia striiformis* f. sp. *hordei (Psh)*, with no similar sequence identified in other fungal species (BlastP, *E*-value < 1 × 10^−5^). Next, to investigate whether PST130_P495001 has structural homology with other fungal effector proteins, we searched the AlphaFold database^29^. Structural and sequence homology searches, using MMseqs2 and Foldseek, clustered PST130_P495001 with twelve predicted effector proteins from *Puccinia graminis* f. sp. *tritici* and two from *Puccinia striiformis* f. sp. *hordei* (Supplementary Table 6 and Supplementary Fig. 7). All proteins in this group are characterised by two anti-parallel β-sheets in their core and a C-terminal α-helix, with most also displaying an N-terminal helical domain (Fig. 6a, b). Furthermore, the secondary structure of PST130_P495001 resembled that of other characterised Avr effectors such as *Pgt* AvrSr50^30^ (Fig. 6c) and *Magnaporthe* AVRs and ToxB-like (MAX) effectors^31^, that also frequently contain two anti-parallel β-sheets and a C-terminal α-helix^32^. These results further support the potential function of PST130_P495001 as a candidate Avr effector protein.

To substantiate the link between low *PST130_P495001* expression and infection of the Kalmar variety, we conducted host-induced gene silencing (HIGS) using a Benchmark-derived *Pst* isolate (DK229_19) that displays regular *PST130_P495001* expression and is avirulent on Kalmar. We inoculated wheat plants of the Kalmar or Benchmark variety with the barley stripe mosaic virus (BSMV) independently expressing two fragments of the *PST130_P495001* gene (Supplementary Fig. 8a) or *BSMV::msc4D*, which contains an artificial sequence not present in wheat or *Pst*, as a viral infection control^33^. At 7–11 days post-viral inoculation (dpvi), plants were inoculated with *Pst* isolate DK229_19 and the efficiency of *PST130_P495001* silencing assessed at 4 dpi by RT-qPCR. A significant decrease in *PST130_P495001* expression was found using both *PST130_P495001* silencing fragments during *Pst* infection of the Kalmar variety (log2 fold change of −2.5 and −2.7; Fig. 6d). Next, we used RT-qPCR to examine the level of fungal biomass present at 4 dpi, revealing a substantial significant log2 fold increase of 2.2 to 2.9 in fungal biomass during *PST130_P495001* silencing and infection of the Kalmar variety (Fig. 6e and Supplementary Data 9). In contrast, during infection of the Benchmark variety only the second *PST130_P495001* fragment induced *PST130_P495001* silencing (log2 fold change of −2.3; Supplementary Fig. 8b), which led to only a minor increase in fungal biomass (log2 fold change of 1.0; Supplementary Fig. 8c). This analysis indicates that suppression of *PST130_P495001* expression can act to markedly enhance *Pst* infection of the Kalmar variety.

## Discussion

Fungal plant disease epidemics are on the rise, driven by an increase in the emergence or influx of new virulent pathogen strains that can trigger sudden, widespread outbreaks as they overcome resistance present in a region’s dominant crop varieties^34^. For instance, in 2010/11, the introduction of a new *Pst* race in Ethiopia, virulent on two widely cultivated wheat varieties Kubsa and Galama, triggered the country’s worst *Pst* epidemic in recent history, causing economic losses exceeding US$250 million^35^. Around the same time, Europe experienced a shift in the *Pst* population starting around 2011, leading to widespread disease outbreaks driven by the introduction of the highly virulent Warrior (*PstS7*) and Warrior(−) (*PstS10*) races^11^. In this study, through genotypic and pathology analysis we confirmed earlier suppositions that the subsequent emergence of the now dominant Kalmar, Amboise and Benchmark *Pst* races^12^, likely resulted from diversification within the *PstS10* race group. The key distinction of these *Pst* isolates, compared to *PstS10* isolates, is their additional virulence on one or more of the Kalmar, Amboise, and Benchmark varieties. Furthermore, we discovered that the recent gain of virulence in the Kalmar and Amboise *Pst* isolates appears associated with expression polymorphism in a single gene, *PST130_P495001*, which encodes a protein with all the typical characteristics of an effector.

Through analysis of *PST130_P495001* expression in *Pst*-infected wheat field samples, we identified a potential association between lower median expression of *PST130_P495001* and infection of wheat varieties sharing a parental lineage with the varieties Claire, Wasp and Robigus. Furthermore, *Pst* infection assays revealed that high levels of infection were only achieved when these varieties were infected with the Kalmar-derived *Pst* isolate, which lacks *PST130_P495001* expression, but not with the Benchmark *Pst* isolate, which retains normal expression levels. These findings strongly suggest PST130_P495001 as a promising candidate for an Avr protein, with the corresponding *R*-gene likely inherited in these varieties through their shared parentage. This was further supported through HIGS experiments, which illustrated that artificial suppression of *PST130_P495001* expression alone can substantially enhance *Pst* infection of the Kalmar variety for a *Pst* isolate usually avirulent on Kalmar. The observed increase in fitness following HIGS in the Kalmar variety suggests that *PST130_P495001* likely encodes an effector and/or a pathogen-derived elicitor that can equally lead to avirulence. However, confirming the avirulence function of candidate *Pst* effectors remains challenging, largely due to the difficulties of validating such functions in obligate biotrophic pathogens like the wheat rust fungi that remain incalcitrant to transformation^36^. In future, cloning the corresponding *R*-gene, likely within the Claire lineage, could help validate the potential avirulence function of PST130_P495001 through pairwise transient co-expression in surrogate systems such as *Nicotiana benthamiana* or co-delivery into wheat protoplasts^37^.

Although no *Avr* genes have been defined for *Pst*, the virulence functions of several *Pst* candidate effectors have been characterised. For instance, Pst_4, and Pst_5 appear to contribute to virulence by interacting with a wheat iron-sulphur protein (*TaISP*) of the cytochrome b6-f complex. This interaction prevents TaISP’s translocation to chloroplasts and, consequently, disrupting its role in chloroplast-derived ROS production^38^. The *Pst* effector Hasp98 promotes virulence by interacting with the wheat mitogen-activated protein kinase 4 (TaMAPK4), a positive regulator of *Pst* resistance^39^. The prior durable resistance reported for the winter wheat cultivar Claire and its derivatives^40^ could reflect the pathogen’s difficulty in modifying *PST130_P495001*, suggesting a potential role as a core virulence factor^41^. This hypothesis is further supported by the remarkably high sequence conservation of *PST130_P495001*. Among the 273 global *Pst* isolates analysed, only 10 exhibited non-synonymous substitutions, with just six *Pst* isolates being homokaryotic for the variation. However, it is unclear how the Kalmar and Amboise *Pst* isolates thrive without expressing a seemingly core virulence factor (*PST130_P495001*). This could potentially be due to recent evolution of functional redundancy among effectors in these *Pst* isolates, which remains to be explored.

Expression polymorphisms are one of the strategies pathogens employ to alter Avr effectors to evade host immunity, as previously observed in the related wheat stem and leaf rust pathogens. For example, the *Pgt AvrSr27* gene exists in at least three known sequence-conserved variants. However, the low expression levels of the virulent *avrSr27-3* allele on chromosome 2A are believed to fall below the threshold needed to trigger an avirulence phenotype, thereby allowing it to evade *Sr27* recognition^17^. Similarly, the sequence-conserved *Pt AvrLr21* gene shows 2-fold higher expression in *Lr21*-avirulent isolates compared to *Lr21*-virulent isolates, enabling them to evade *Lr21* recognition^18^. In the case of *PST130_P495001*, its absence of expression in Kalmar and Amboise *Pst* isolates is exceptionally rare, observed in only 11 out of 888 additional *Pst* datasets assessed. Further examination of the genomic region surrounding *PST130_P495001* revealed complete conservation between the Kalmar, Amboise and Benchmark *Pst* isolates in the 2 Kb upstream and downstream regions of the *PST130_P495001* locus. This finding suggests that expression regulation is unlikely to be mediated by changes in transcriptional regulators of *PST130_P495001*. Alternatively, we speculate that epigenetic silencing of *PST130_P495001* expression could instead play a role in regulating virulence in Kalmar and Amboise *Pst* isolates.

The critical contribution of epigenetics in virulence gains for human pathogens is well established^42^. For filamentous plant pathogens, examples are limited, with the best examples for *Phytophthora sojae*, the oomycete responsible for soybean stem and root rot^43^. In *P. sojae* transgenerational gene silencing of *Avr3a* has been linked to enhanced virulence against *Rps3a*^44^, and transcriptional polymorphisms in *Avr1b* have been associated with evasion of *Rps1b* resistance^45^. Similarly, epigenetic switching of *PST130_P495001* expression could provide a versatile and reversible means for regulating expression states, contributing to the recent gain of virulence on the Kalmar and Amboise and related wheat varieties with similar resistance specificities. However, the exact mechanism governing loss of *PST130_P495001* expression remains to be determined.

Advances in fungal genomics have significantly accelerated the identification of potential virulence loci, with hundreds of candidate effectors proposed for many agriculturally important fungal pathogens^46,47^. Although systems for confirming Avr effector function in wheat remain limited and challenging, the validation of the first five *Pgt* and two *Pt* Avr effectors is promising^15–18,37,48^. This progress has already demonstrated that wheat rust fungi employ a variety of mechanisms to achieve virulence gains, with this study further emphasising the importance of expression polymorphisms in the case of the candidate *PST130_P495001 Avr* effector. In the future, this knowledge could improve the monitoring of host recognition loss and contribute to the development of more effective and sustainable resistance deployment strategies, ultimately helping mitigate the severe crop losses currently caused by these fungal parasites.

## Materials and methods

### *Pst* infection assays

*Pst*-infected wheat leaves from Kalmar (Nordic Seed, Denmark), Amboise (Blackman Agricultural Ltd, UK), and Benchmark (Sejet Plant Breeding, Denmark) wheat varieties (isolates DK267_17, BE166_20, DK229_19) were kept on moist filter papers in petri dishes at 15 °C for 1–2 days to promote urediniospore formation. Fresh uredinospores were then used to inoculate the susceptible wheat line Morocco by gently rubbing infected leaf segments onto the abaxial side of 2-week-old seedling leaves. The inoculated seedlings were misted with water and incubated in darkness at 10 °C for 24 h under high relative humidity before being transferred to greenhouse cabins maintained at 17 °C (day)/12 °C (night), with a 16-h photoperiod of natural light supplemented with sodium light (100 μmol s⁻¹ m⁻²) and 8 h dark. Urediniospores were harvested at approximately 15 dpi. Subsequently, two-week-old seedlings were inoculated with 10 mg of *Pst* urediniospores, resuspended in Novec 7100 (1 mg/mL, Sigma-Aldrich, St. Louis, MO, USA), and applied using an airbrush spray gun^49^. Each *Pst* isolate was used to independently inoculate a set of 37 wheat differential lines alongside Kalmar, Amboise, and Benchmark wheat varieties. This differential set enabled detection of virulence to 19 yellow rust resistance genes: *Yr1, Yr2, Yr3, Yr4, Yr5, Yr6, Yr7, Yr8, Yr9, Yr10, Yr15, Yr17, Yr24, Yr25, Yr27, Yr32*, as well as *YrSp* (Spaldings Prolific), *YrAvS* (Avocet S), *YrAmb* (Ambition). Virulence profiles were assessed on both first and second leaves, where infection types (IT) 0–6 indicated avirulence (incompatibility) and IT 7–9 indicated virulence (compatibility)^50^. Virulence to individual *Yr* genes was inferred based on infection types across two to three differential lines, and each unique virulence phenotype was classified as a distinct race. In addition, the Kalmar (DK267_17) and Benchmark (DK229_19) *Pst* isolates were separately used to inoculate an additional panel of 11 commercial wheat varieties (Supplementary Table 6) using spray inoculation as described above, with disease severity assessed 16 dpi.

### RNA-seq of *Pst*-infected wheat leaf samples

Total RNA was extracted from the leaves of the wheat variety Morocco at 14 dpi following infection with the *Pst* isolates originally identified on the Kalmar (6 isolates), Amboise (4 isolates) and Benchmark (7 isolates) varieties using a Qiagen RNeasy Mini Kit (Qiagen, Hilden, Germany). Total RNA was analysed for quality and quantity using a Qubit fluorometer (Thermo Fisher Scientific, Waltham, Massachusetts, USA), cDNA libraries prepared using an Illumina TruSeq RNA Sample Preparation Kit (Illumina, San Diego, California, USA) and sequencing conducted on an Illumina NovaSeq instrument by Azenta Life Sciences (Burlington, Massachusetts, USA). The resulting 150-bp paired-end reads were filtered for quality using default parameters and trimmed to remove the first 13 nucleotides that may contain potential remaining adaptor sequences using FASTX-Toolkit version 0.0.13.2.

### Phylogenetic analysis of *Pst* isolates

In addition to the 17 RNA-seq datasets generated above, genomic, or RNA-seq datasets for a total of 416 *Pst* isolates collected across 29 countries were obtained from public repositories (Supplementary Data 1). Each dataset was independently aligned to the *Pst* reference genome (*Pst* isolate 104E137A-^19^; GenBank Assembly ID: GCA_002900275.1) using BWA v. 0.7.5^51^ for genomic DNA samples and STAR v.2.5^52^ for RNAseq datasets, with transcriptomic reads trimmed at splice junctions using the SplitNCigarReads tool in GATK version 4.0^53^. Variant calling was conducted using SAMtools v.0.1.1.9^51^. BAM files were processed with the SAMtools mpileup function using the reference genome from *Pst* isolate 104E137A-^19^ to generate pileup-format data. SNPs meeting a minimum depth of coverage (10x for genomic DNA and 20× for RNA-seq) were identified and used for downstream analysis^54^. Phylogenetic analysis was performed using synthetic gene sets for each *Pst* isolate that incorporated the variation identified above and was restricted to the third codon position for genes with a minimum of 80% breadth of coverage for all samples^55^. The phylogeny was generated using an approximate maximum likelihood approach and generalised time-reversible model of nucleotide evolution in FastTree version 2.1.11^56^ and visualised in iTol version 7^57^. Two further phylogenetic trees were generated to assess the genetic similarity of *Pst* isolates lacking *PST130_P495001* expression following the method above and including additional RNA-seq datasets from *Pst* isolates lacking *PST130_P495001* expression with or without the one *Pst* isolate from *T. durum*, alongside 423 additional *Pst* isolates to place the isolates in a global context.

### Assessing differential gene expression and genetic diversity between *Pst* isolates

Transcript abundance was assessed for each of the 17 *Pst* isolates from Kalmar, Amboise and Benchmark using pseudoalignments to the *Pst* reference genome available in the Ensembl BioMart database (isolate PST-130^20,21^; GenBank Assembly ID: GCA_000223505.1) generated with Kallisto version 0.4.6.1 and default parameters^58^. Pairwise comparisons of normalised transcript abundance were performed between *Pst* isolates from Kalmar and/or Amboise to those from the Benchmark variety, with genes considered significantly differentially expressed having an adjusted *p-*value of <0.05 and absolute log_2_ fold change greater than or less than 2. The distribution of differentially expressed genes (DEGs) was visualised using plots generated with the Sleuth R package^59^ to illustrate the relationship between mean expression and log_2_ fold change. Gene expression data was modelled using a linear model framework, where a full model was fit to test for differences in expression between *Pst* isolates found on two wheat varieties (Benchmark vs. Amboise or Kalmar), and a reduced model that does not account for the group differences. To identify DEGs, we compared the two models using a likelihood ratio test. In addition to this, we employed the Wald test to estimate the effect size (log_2_ fold change) and test whether the coefficient associated with the two groups was significantly different from 0. To assess the genetic variation between the Kalmar, Benchmark, and Amboise *Pst* isolates, the Weir and Cockerham fixation index (*F*_*ST*_) was calculated for each SNP using VCFtools version 1.2^60^. Resulting *F*_*ST*_ values were averaged across all SNPs to obtain a genome-wide estimate of genetic differentiation between the three *Pst* isolate groups.

### Genome sequencing of *Pst* isolates

A single representative isolate from each of the Benchmark, Kalmar and Amboise *Pst* isolate groups (Supplementary Data 1) was selected for full genome sequencing: DK267_17 (*cv*. Kalmar), BE166_20 (*cv*. Amboise) and DK229_19 (*cv*. Benchmark). Genomic DNA was extracted from ~80 mg of dried urediniospores using a previously established cetyltrimethylammonium bromide (CTAB) method^61^. *Pst* urediniospores were combined with sterile sand (50–70 mesh particle size; Sigma-Aldrich, St. Louis, MO, USA) and ground into a fine powder using a pestle and mortar over dry ice. Subsequently, 2 ml of prewarmed CTAB extraction buffer (50 °C; 137 mM d-sorbitol, 34 mM N-lauroylsarcosine sodium salt, 24 mM centrimide, 0.8 M NaCl, 22 mM EDTA, 10 g/L polyvinylpolypyrrolidone) and 5 µl proteinase K (20 mg/ml; Thermo Fisher Scientific, Waltham, Massachusetts, USA) were added. The mixtures were incubated at 50 °C for 2 h, after which one volume of chloroform:isoamyl alcohol (24:1; Thermo Fisher Scientific, Waltham, Massachusetts, USA) was added and samples were centrifuged at 1922 × *g* for 10 min. The aqueous phase was transferred to a clean tube and treated with 20 µl RNase (10 mg/ml; Thermo Fisher Scientific, Waltham, Massachusetts, USA), followed by incubation at room temperature for 1 h. A second chloroform:isoamyl alcohol extraction was performed and the samples centrifuged. The resulting aqueous phase was transferred to a clean tube, and DNA was precipitated by adding one volume of isopropanol. Samples were incubated at −20 °C overnight and centrifuged at 1922 × *g* for 10 min, after which supernatant was discarded. The DNA pellets were rinsed twice with 70% ethanol, air dried for 30 min, and finally resuspended in 30 µl TE buffer (10 mM Tris-HCl, 1 mM EDTA, pH 8.0).

The quality and quantity of gDNA obtained assessed using a Qubit fluorometer (Thermo Fisher Scientific, Waltham, Massachusetts, USA). gDNA libraries were prepared using the Nanopore ligation sequencing kit (SQK-LSK110; Oxford Nanopore Technologies, Oxford, UK), following the manufacturer’s instructions and sequenced on the Oxford Nanopore MinION platform with R9.4.1 flow cells (Mk1B; Oxford Nanopore Technologies, Oxford, UK). Subsequent base-calling was performed using Guppy v.6.0.6^62^ with default parameters and reads trimmed using NanoFilt v2.8.0^63^ to remove reads less than 500 bases in length or with quality scores <10 and to remove the first 20 bases from all reads. Trimmed reads were aligned to the *Pst* chromosome-level reference genome (isolate 134E16A + 17 + 33 + ^19^; GenBank Assembly ID: GCF_021901695.1) using minimap2 (v.2.24^64^) and SAMtools (v1.18^51^). The *PST130_P495001* genomic locus, including 2 Kb upstream and downstream of the *PST130_P495001* gene, was reconstructed from sequence alignments of each *Pst* isolate to the *Pst* reference genome (isolate 134E16A + 17 + 33 + ^19^) and aligned for visualisation locally using the EMBL-EBI Job Dispatcher website and MUSCLE version 3.8.31^65^. Flanking regions (20 Kb) surrounding the *PST130_P495001* CDS or four housekeeping genes (*Elongation Factor 1*, *Beta-tubulin*, *Alpha-tubulin* and *Actin*) were extracted from the *Pst* reference genome (isolate 134E16A + 17 + 33 + ^19^) and annotated with repeat elements using RepeatMasker version 4.1.2^66^.

### Annotation and assessment of *PST130_P495001* sequence diversity

PST130_P495001 was used in a sequence similarity search against the NCBI non-redundant protein database. The resulting fifteen high-confidence protein hits (*e*-value < 1e-5) from *Psh* and *Pgt* (Supplementary Table 6) and PST130_P495001 were aligned using Clustal version 2.1 and a phylogenetic tree generated using FastTree version 2.1.11^56^ and visualised using iTol version 7^57^. The predicted protein sequence of *PST130_P495001* (XP_047806342.1) and the aforementioned fifteen proteins from *Psh* and *Pgt* were assessed for the presence of a secretion signal using SignalP 6.0 with the “Eukarya” database^22^ and analysed using EffectorP-fungi 3.0^24^ to estimate the likelihood of functioning as cytoplasmic or apoplastic effectors. The predicted monomeric protein structure of PST130_P495001 was obtained from the AlphaFold Protein Structure Database (UniprotID: A0A2S4WAX4), using the AlphaFold2 algorithm^67^. MMseqs2^68^ and Foldseek^69^ were used to identify proteins with similar structural domains. The *Pgt* AvrSr50 protein structure was obtained from an X-ray diffraction structure (PDB ID: 7mqq^30^). Root-mean-squared (RMSD) values from superimposed protein structures with similar structures, were determined using MatchMaker in ChimeraX version 1.10. The RMSD value reflected the difference in the position of the aligned backbone C’s (Cα) and ‘pruned’ atom pairs were obtained by excluding ‘outlier’ residue pairs where the pairwise distances were too large.

Sequence diversity in the *PST130_P495001* locus was examined using publicly available data for 416 global *Pst* isolates that were collected across 29 countries (Supplementary Data 1). Each dataset was aligned to the *Pst* reference genome (isolate 104E137A-^19^) using STAR v.2.5^52^ for transcriptomic reads, BWA v. 0.7.5^51^ for genomic reads, and variant calling conducted using SAMtools v.0.1.1.9^51^. *Pst* isolates with ambiguities in the *PST130_P495001* sequence were removed, resulting in 273 datasets. The average number of SNPs/base in the *PST130_P495001* gene was assessed for each dataset by comparison to the *Pst* reference sequence (isolate 104E137A-^19^) and PST130_P495001 isoforms defined. In addition, the SNPs/base of three highly conserved genes for comparison were determined using subsets of the 273 *Pst* isolates devoid of any ambiguity in each respective gene: *Actin* (*PST104_9243*), *Beta-tubulin* (*PST104_20937*) and *Elongation Factor 1* (*PST104_11781*). Further analysis of the *PST130_P495001* sequence was conducted using the phased *Pst* reference genome assembly (*Pst* isolate 104E137A-^19^) and 19 further *Pst* genome assemblies (Supplementary Table 3). The *PST130_P495001* sequence was identified in each genome assembly through BLAST searches, the corresponding gene sequence extracted, and alignments were done locally on the EMBL-EBI Job Dispatcher website using MUSCLE version 3.8.31^65^. In addition, gene sequences for 156 genes that were previously shown to display a similar expression profile as *PST130_P495001*^19^ were compiled and the SNPs per base rate across 273 global *Pst* isolates assessed.

### Evaluation of *PST130_P495001* expression

Publicly available *Pst* gene expression data^27^ was extracted, and *PST130_P495001* expression assessed from the transcript counts, across 992 available RNAseq datasets that included an array of *Pst* developmental stages: isolated *Pst* haustoria, ungerminated urediniospores, germinated urediniospores and *Pst*-infected wheat leaf tissue (Supplementary Data 7). To determine if any of the datasets from *Pst*-infected wheat leaf tissue lacked *PST130_P495001* expression, similar to the Kalmar and Amboise *Pst* isolates, first the general coverage of the datasets was examined to remove those with low coverage that could skew the results. Accordingly, transcript counts for four housekeeping genes were analysed: *Actin* (*PST130_09225*), *Alpha-tubulin* (*PST130_10193*)*, Beta-tubulin* (*PST130_06515*), and *Elongation factor 1* (*PST130_14067*). Datasets with transcript counts below 15% of the median expression level for these housekeeping genes were excluded, resulting in 888 datasets being retained for further analysis. Transcript counts were then examined for *PST130_P495001* in each of the 888 datasets and those with zero counts for *PST130_P495001* were classified as lacking *PST130_P495001* expression.

### Yeast signal sequence trap system

To confirm the function of the predicted *PST130_P495001* secretion signal, we employed the yeast signal sequence trap system using the pSUC2T7M13ORI (pSUC2) vector, which contains a truncated SUC2 invertase gene, lacking both the initiation methionine and signal peptide^70^. A 60 bp fragment including the predicted *PST130_P495001* secretion signal (PST130_P495001^1–20^) was synthesised and cloned into the pSUC2 vector^23^ in frame with the invertase gene by Azenta Life Sciences (Burlington, Massachusetts, USA). The invertase secretion-deficient *Saccharomyces cerevisiae* strain YTK12 was transformed with the pSUC2, pSUC2-PST130_P495001^1–20^ or the pSUC2-PexRD8^1–24^ positive control^23^ using the lithium acetate method^71^. In brief, the YTK12 strain was cultured overnight at 30 °C in YPD media ((1% yeast extract, 2% peptone, 2% glucose). The culture was adjusted to an OD_600_ of 0.1 and incubated at 30 °C for 4–6 h. Cells were pelleted by centrifugation at 1768 × *g* and resuspended in 1x LiOAc buffer (0.1 M LiOAc, 10 mM Tris-HCl (pH 8.0)). A 100 μl aliquot of resuspended cells was mixed with 10 μl of UltraPure^TM^ salmon sperm carrier DNA (Thermo Fisher Scientific, Massachusetts, USA) and 1 μg of each plasmid independently. Each sample was then combined with 280 μl of 50% polyethylene glycol 3350 and incubated at room temperature for 45 min. Dimethyl sulfoxide (43 µl) was added to each sample, and the mixture vortexed before incubation at 42 °C for 10 min, followed by chilling on ice for 3 min. The cells were centrifuged at 883 × *g*, washed in distilled water and then resuspended in 200 µl of TE buffer. Following transformation, yeast was plated on YPDA media (1% yeast extract, 2% peptone, 2% glucose, 0.003% adenine hemisulfate and 2% agar) and incubated at 30 °C for 2–3 days, with positive clones confirmed by PCR using vector (Forward: 5’-GGTGTGAAGTGGACCAAAGGTCTA-3’) and *PST130_P495001* (Reverse: 5’-TCCTGCACAAGCTGTG-3’) specific primers. Positive clones were grown on CMD-W (0.67% yeast N base without amino acids, 0.075% tryptophan dropout supplement, 2% sucrose, 0.1% glucose, and 2% agar) plates or restrictive media with raffinose as the sole carbon source (YPRAA media: 1% yeast extract, 2% peptone, 2% raffinose, 2 μg/ml antimycin A and 2% agar) to assay for invertase secretion. Plates were incubated at 30 °C for 3–4 days. Invertase enzymatic activity was analysed through assessment of reduction of 2,3,5-triphenyltetrazolium chloride (TTC: Sigma-Aldrich, Merck KGaA, Darmstadt, German) to insoluble red coloured triphenylformazan^72^. YTK12 strains were mixed with 1 mL of buffered sucrose (25 mM sucrose in 0.2 M NaAc, pH 5.0) and incubated at 37 °C for 30 min. An equal volume of 2,3,5-triphenyltetrazolium chloride (TTC, 0.2% TTC in 4% NaOH) was then added to each sample, and the resulting colorimetric change observed after a 5 min incubation at room temperature.

### Subcellular localisation of PST130_P495001

Seedlings of the Vuka variety were infected with *Pst* 13/14 isolate^54^ as described above. RNA was extracted using the RNeasy Plant mini kit (Qiagen, Hilden, Germany), and cDNA synthesised using the Verso cDNA Synthesis Kit (Thermo Fisher Scientific, Waltham, Massachusetts, USA). The resulting cDNA was used to amplify the *PST130_P495001* CDS (438 bp) lacking the region predicted to encode a signal peptide (1–57 bp) with the addition of CACC to facilitate Gateway cloning using primers: forward 5’-CACCGGATTGATTTCAACCATCACACATGAGAAC-3’ and reverse 5’TTAATCTCGCAAAGAGCGAAGAAAAGTCATTA-3’. The amplified *PST130_P495001*^*58-438*^ fragment was cloned into the pK7WGF2 vector^73^ using the Gateway cloning system (Invitrogen, Waltham, Massachusetts, USA). The resulting N-terminal GFP fusion construct (GFP:PST130_P495001), along with the empty vector control (GFP) was introduced into *Agrobacterium tumefaciens* strain GV3101. *A. tumefaciens* cells were thawed on ice, mixed with 1μg of plasmid DNA and incubated on ice for 30 min. Samples were then frozen in liquid nitrogen for 5 min, incubated at 37 °C for 5 min and placed back on ice for 5 min. Transformed cells were grown at 28 °C for 48 h and resuspended at an OD_600_ of 0.5 in infiltration buffer (10 mM MgCl2, 10 mM MES-K (pH 5.6), 100 μM acetosyringone). The suspensions were incubated at room temperature with gentle agitation for 3 h before being used to infiltrate leaves of 4-week-old *Nicotiana benthamiana* plants using a syringe^74^. After 3 days, approximately 1 cm^2^ sections of *N. benthamiana* leaves were cut and mounted in water. Slides were imaged on a Zeiss LSM880 Airyscan confocal microscope using 488 nm (25 mW Argon Ion laser) excitation with emission bandwidth of 495–550 nm. The SR-option of the Airyscan Fast mode was used with a Plan Apochromat 40×/NA 1.1 water-immersion objective and single planes were taken with pixel dimensions of 54 × 54 nm followed by 2D Airyscan processing with default settings (Zeiss, Germany).

### Evaluation of host wheat variety parentage

A total of 321 publicly available *Pst*-infected RNA-seq datasets were collated, where the wheat variety was noted, and four or more *Pst-*infected samples were collected^27^. Transcript counts were analysed for each dataset and the median value for *PST130_P495001* expression determined. *Pst* isolates with statistically lower *PST130_P495001* expression than the overall population median (*padj* < 0.4 and abs(log2(Fold Change) > 0.75) were determined using the limma variance modelling at the observational level (voom) pipeline^75^ and classified as “low *PST130_P495001* expression”. The parental lineage of each of the 20 *Pst*-infected wheat varieties assessed was determined through analysis of an available wheat pedigree dataset^28^. A network of 1st and 2nd generations was generated using Helium 2.1.0^76^.

### Host-induced gene silencing (HIGS) and reverse transcription quantitative PCR (RT-qPCR)

Two fragments of the *PST130_P495001* gene (171-bp and 151-bp) were selected for HIGS using the siRNA-Finder software^77^. Each fragment was synthesised by Genewiz (Azenta Life Sciences, Massachusetts, USA) and cloned into the BSMV vector pCa-γbLIC^78^. *BSMV::TaPDS* was used as a positive control in wheat and *BSMV::msc4D* as a viral infection control^33^. Each construct was transformed independently into *Agrobacterium tumefaciens* strain GV3101 and transiently expressed in *Nicotiana benthamiana* leaves^74^ as described above. Once *BSMV*-induced viral mosaic symptoms were visible approximately 7–12 dpvi, sap was harvested from *N. benthamiana* leaves and used to inoculate approximately 14-day-old wheat seedlings (*cv*. Kalmar and Benchmark) grown in a glasshouse under long-day conditions (16-h-light/8-h-dark photoperiod and 19 °C/14 °C cycles) using mechanical abrasion. Plants were immediately transferred to a controlled environment cabinet (MLR-352-PE, PHCbi, Panasonic Corporation, Kadoma, Japan) with a 16-h-light/8-h-dark photoperiod and 23 °C/16 °C cycles with LED fluorescent tubes (Fusion Lamps, Doncaster, UK). Once the *BSMV::PDS* photobleaching phenotype was visible (7–11 dpvi), wheat seedlings were spray inoculated with *Pst* urediniospores from the Benchmark-derived isolate (DK229_19) resuspended in Novec 7100 as described above.

*Pst*-infected wheat leaf samples were collected 4 dpi, total RNA extracted from ~100 mg of tissue using a Qiagen Plant RNeasy Mini kit (Qiagen, Hilden, Germany) and residual genomic DNA removed using the TURBO DNA-free™ Kit (Thermo Fisher Scientific, Massachusetts, USA). First-strand cDNA was synthesised using the Verso cDNA synthesis kit (Thermo Fisher Scientific, Massachusetts, USA) with Oligo(dT) following the manufacturer’s instructions. qPCR was performed using LightCycler 480 SYBR Green I Master Mix (Roche, Basel, Switzerland) following the manufacturer’s instructions, using primers at a final concentration of 0.25 µM. Silencing was confirmed by comparing the expression of *PST130_P495001* to the *PstEF1* gene^79^ and analysis of fungal biomass was conducted by comparing the expression of the fungal *PstEF1* gene^79^ and wheat *TaUCE* gene^80^. Each experiment was conducted with two to five biological replicates (separate plants) and three technical replicates per plant.

### Statistics and reproducibility

Virulence profiles were assessed for *Pst* isolates identified on Kalmar, Amboise and Benchmark across different wheat varieties using two to four biological replicates. Transcript abundance was compared between *Pst* isolates from Kalmar (6 isolates) and/or Amboise (4 isolates) against *Pst* isolates from Benchmark (7 isolates) using the Benjamini-Hochberg method in the Ensembl BioMart database, with genes considered significantly differentially expressed having an adjusted *p-*value of <0.05 and absolute log_2_ fold change greater than or less than 2. We employed the Wald test to estimate the effect size (log_2_ fold change) and to test whether the coefficient associated with the two groups was significantly different from 0. Assessment of 321 publicly available *Pst*-infected RNA-seq datasets was conducted to identify *Pst* isolates with statistically lower *PST130_P495001* expression than the overall population median (*padj* < 0.4 and abs(log2(Fold Change) > 0.75) using the limma variance modelling at the observational level (voom) pipeline^75^. Following HIGS of *PST130_P495001*, RT-qPCR data were examined using a two-tailed Student’s *t* test to assess successful *PST130_P495001* silencing and changes in *PstEF1* expression to examine changes in fungal biomass. Each HIGS assay was conducted with two to five biological replicates (separate plants) and three technical replicates per plant. All statistical tests were conducted in R and Excel, and all available samples were used in the study. Sample size and repetition in each experiment was largely determined by availability of material.

### Reporting summary

Further information on research design is available in the Nature Portfolio Reporting Summary linked to this article.

## Supplementary information

Supplementary Information

Description of Additional Supplementary Files

Supplementary Data 1-10

Reporting summary

## Data Availability

Sequence data that supports the findings of this study has been deposited in the Sequence Read Archive (SRA: PRJNA1231252) and additional data is available in the supplementary material of this article (Supplementary Tables 1– 6 and Supplementary Data 1–9). The location of datasets used to plot the graphs presented is described in Supplementary Data 10. The vectors pSUC2-PST130_P495001^1–20^ and GFP:PST130_P495001 are available at addgene (IDs: 254450 and 254451, respectively).

## Code availability

All custom computer code and the newick files for the phylogenies are available on GitHub^81^ and zenodo^82^.
