# Supplementary material for: Virulence gains in the Puccinia striiformis f. sp. tritici PstS10 lineage correlate with expression polymorphism in a candidate Avr effector
Source: Commun Biol. 2026 Apr 13;9:790. doi: 10.1038/s42003-026-10018-0 (PMC13254256; doi:10.1038/s42003-026-10018-0)
Supplement: Supplementary file 1 — Supplementary Information [file 42003_2026_10018_MOESM1_ESM.pdf]

COMMUNICATIONS BIOLOGY: Supplementary Information

Title: Virulence gains in the *Puccinia striiformis* f. sp. *tritici PstS10* lineage correlate with expression polymorphism in a candidate Avr effector

Rita Domingues Carvalho, Loizos Savva, Julian Rodriguez Algaba, Andrey Korolev, Christopher Stephens, Anthony Bryan, Annemarie F. Justesen, Mogens S. Hovmøller, Diane G.O. Saunders

The following Supplementary Information is available for this article:

Supplementary Fig. 1. Representative images of infection phenotypes of *Pst* isolates from Kalmar, Amboise and Benchmark on the Benchmark, Kalmar, and Amboise varieties.

Supplementary Fig. 2. Pairwise sequence comparisons of *Pst* isolates from Kalmar, Amboise and Benchmark identified no group-specific gene isoforms linked to the differences in virulence profiles but did reveal significant differential expression of *PST130_P495001* between *Pst* isolates from Amboise and Benchmark.

Supplementary Fig. 3. Sequence alignment of *PST130_P495001* illustrates complete conservation in the coding and 2 Kb flanking regions in Kalmar, Benchmark, and Amboise *Pst* isolates.

Supplementary Fig. 4. Similar repeat elements were identified within 20 Kb regions flanking the *PST130_P495001* locus and four housekeeping genes.

Supplementary Fig. 5. *PST130_P495001* is highly conserved in sequence across *Pst* isolates.

Supplementary Fig. 6. *Pst* isolates lacking *PST130_P495001* expression have arisen independently in several distinct lineages including one containing *Pst* isolates from *Triticum durum*.

Supplementary Fig. 7. Proteins with sequence similarity to PST130_P495001 are limited to *Pst* and *Psh*.

Supplementary Fig. 8. Host-induced gene silencing (HIGS) of *PST130_P495001*.

Supplementary Table 1. Virulence profiling of *Pst* isolates identified on Kalmar, Amboise and Benchmark wheat varieties.

Supplementary Table 2. Assessment of PST130_P495001 for features typical of an effector protein.

Supplementary Table 3. Details of the 19 *Pst* genome assemblies examined in this study.

Supplementary Table 4. Parentage of 20 wheat varieties that were infected with *Pst* isolates with regular or low *PST130_P495001* expression.

Supplementary Table 5. Infection types following inoculation of 11 wheat varieties with *Pst* isolates collected on Kalmar or Benchmark.

Supplementary Table 6. Assessment of proteins from *Psh* and *Pgt* with structural homology to PST130_P495001 for features typical of an effector protein.

Supplementary Data 1 (separate xlsx file). Description of *Pst* isolates analysed in this study.

Supplementary Data 2 (separate xlsx file). Percentage of reads aligned to the *Pst* reference genome assembly (isolate Pst104E137).

Supplementary Data 3 (separate xlsx file). Virulence profiling of *Pst* isolates identified on Kalmar, Amboise and Benchmark on the respective wheat varieties and the standard susceptible line Morocco. Infection types were assessed in replicate on a 0 – 9 scale, with 0-3 considered susceptible, 4-6 intermediate and 7-9 resistant.

Supplementary Data 4 (separate xlsx file). Virulence profiling of *Pst* isolates identified on Kalmar, Amboise and Benchmark wheat varieties. Infection types were assessed in replicate on a 0 – 9 scale, with 0-3 considered susceptible, 4-6 intermediate and 7-9 resistant.

Supplementary Data 5 (separate xlsx file). Pairwise comparisons of gene expression between *Pst* isolates from Amboise or Kalmar to those from Benchmark *Pst* isolates.

Supplementary Data 6 (separate xlsx file). Single nucleotide polymorphisms identified per base in *PST130_P495001*, *Actin*, *Beta-tubulin* and *Elongation Factor 1* for 273 *Pst* isolates analysed.

Supplementary Data 7 (separate xlsx file). Metadata describing the 992 *Pst* RNA-seq datasets extracted from the *Pst* expression browser.

Supplementary Data 8 (separate xlsx file). *PST130_P495001* expression across *Pst*-infected samples derived from 35 wheat varieties where at least four *Pst*-infected samples were available.

Supplementary Data 9 (separate xlsx file). Host-induced gene silencing of *PST130_P495001* and fungal biomass assessed using RT-qPCR at 4 days-post infection (dpi) with *Pst* and 11-15 days post-viral inoculation (dpvi).

Supplementary Data 10 (separate xlsx file). Location of datasets used to generate figures.

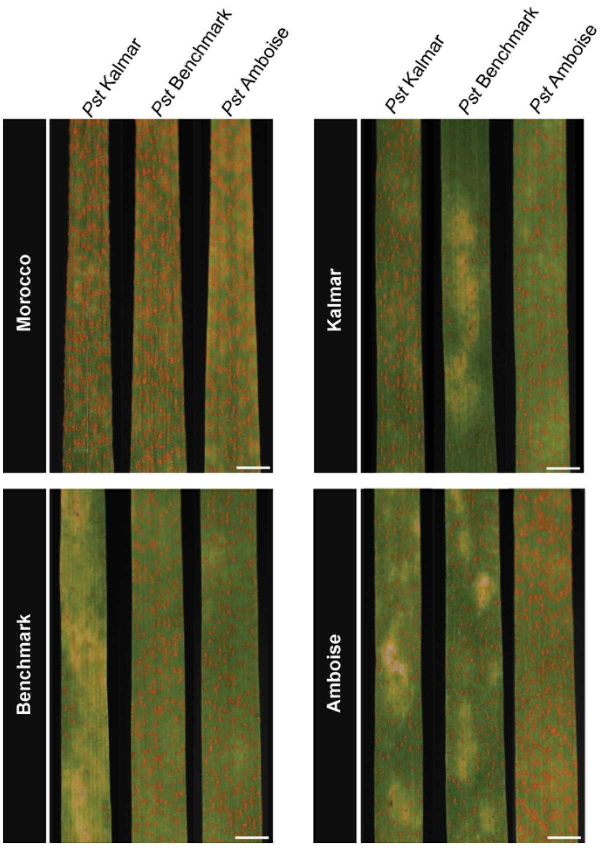

Supplementary Fig. 1. Representative images of infection phenotypes of *Pst* isolates from Kalmar, Amboise and Benchmark on the Benchmark, Kalmar, and Amboise varieties. *Pst* isolates collected on the Benchmark, Kalmar, and Amboise wheat varieties, varied in their virulence profile on Benchmark, Kalmar, and Amboise. Each *Pst* isolate was inoculated onto the three wheat varieties at seedling stage and disease severity assessed approximately 16 days after inoculation. Infection types on the 'Morocco' wheat variety were included as a universally susceptible control. Scale bars represent 5 mm.

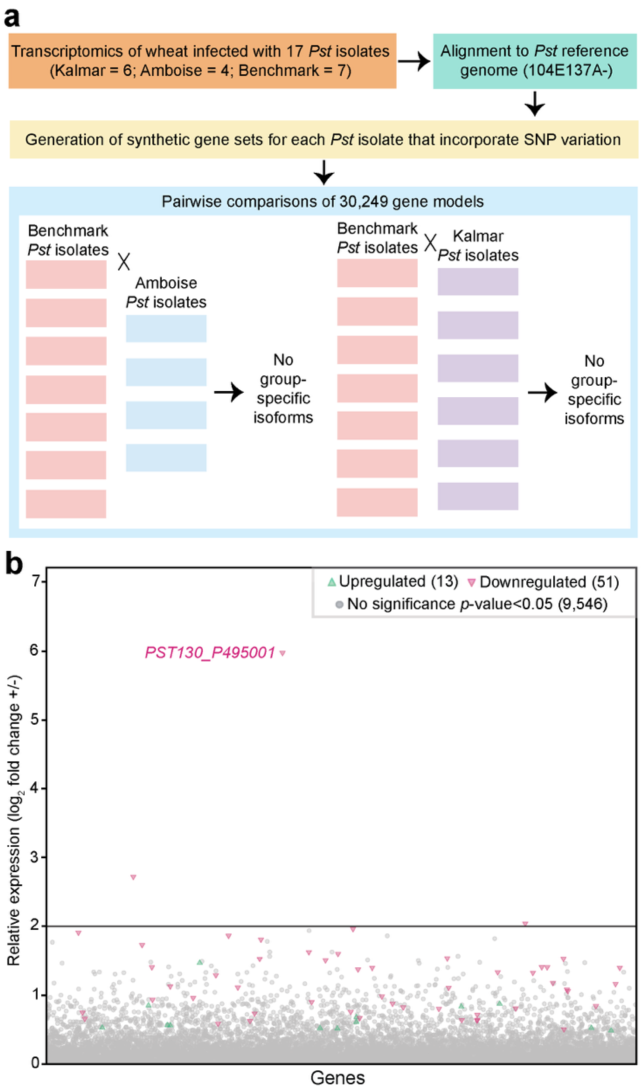

Supplementary Fig. 2. Pairwise sequence comparisons of *Pst* isolates from Kalmar, Amboise and Benchmark identified no group-specific gene isoforms linked to the differences in virulence profiles but did reveal significant differential expression of *PST130_P495001* between *Pst* isolates from Amboise and Benchmark. a) Sequence comparisons were conducted to identify any specific gene isoforms that could explain the differences in virulence profiles between *Pst* isolates from Kalmar (6 isolates) and Amboise (4 isolates) to those from Benchmark (7 isolates). Each dataset was aligned to the *Pst* reference genome (*Pst* isolate 104E137A-[1]) and sequence comparisons between *Pst* groups conducted across 30,249 gene models. **b)** Pairwise comparisons of gene expression profiles between all *Pst* isolates from Amboise (4 isolates) and Benchmark (7 isolates) identified *PST130_P495001* as having the most significant fold change (-5.9730 (p-value $6.1884e^{-52}$)). Assessments of transcript abundance were performed using Kallisto version 0.4.6.1[2], with genes considered

significantly differentially expressed ('Upregulated' and 'Downregulated') having an adjusted *p*-value of < 0.05 and absolute $\log_2$ fold change greater than or less than 2.

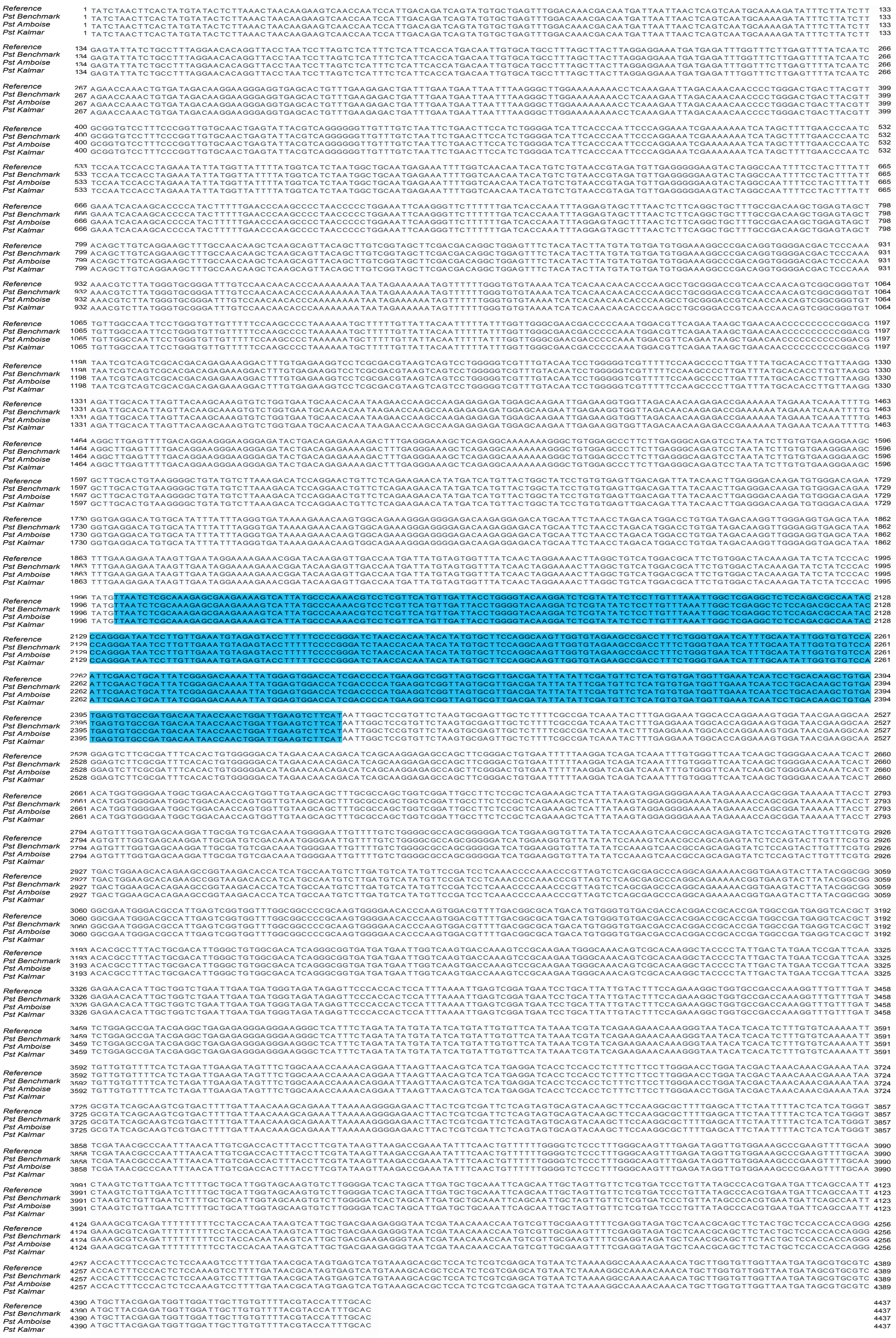

Supplementary Fig. 3. Sequence alignment of *PST130_P495001* illustrates complete conservation in the coding and 2 Kb flanking regions in Kalmar, Benchmark, and Amboise *Pst* isolates. Sequence alignments of the *PST130_P495001* coding sequence and 2 Kb upstream and downstream regions for one *Pst* isolate each from Kalmar, Amboise and

Benchmark were conducted locally using the EMBL-EBI Job Dispatcher website and MUSCLE version 3.8.31[3]. *PST130_P495001*, light blue background in reverse orientation.

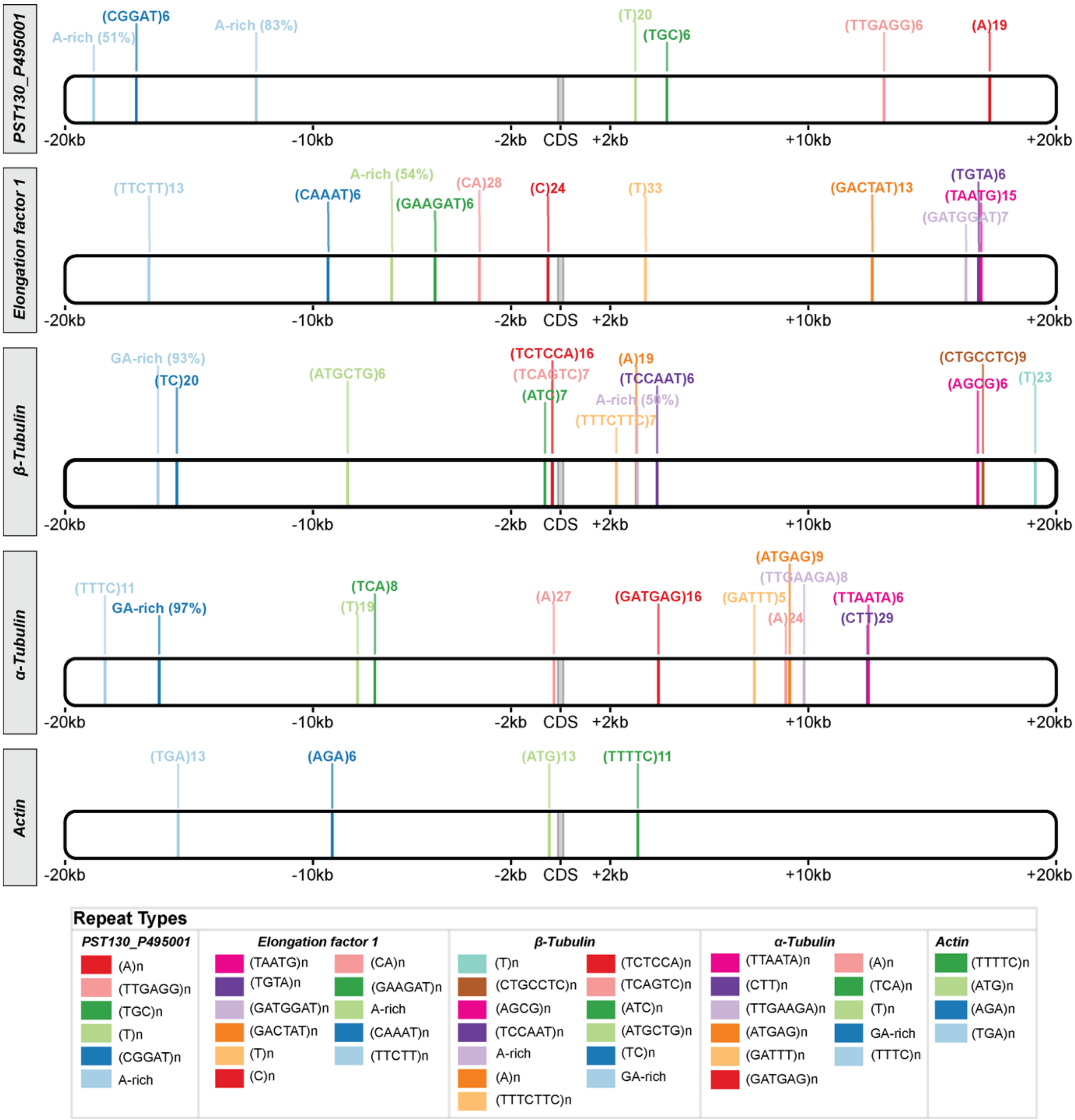

Supplementary Fig. 4. Similar repeat elements were identified within 20 Kb regions flanking the *PST130_P495001* locus and four housekeeping genes. Repeat elements annotated within 20 Kb flanking regions of the *PST130_P495001* coding sequence (CDS) were also identified within 20 Kb regions surrounding the four housekeeping genes: *Elongation Factor 1*, *Beta-tubulin*, *Alpha-tubulin* and *Actin* (isolate 134E16A+17+33+[1]) that are invariably expressed.

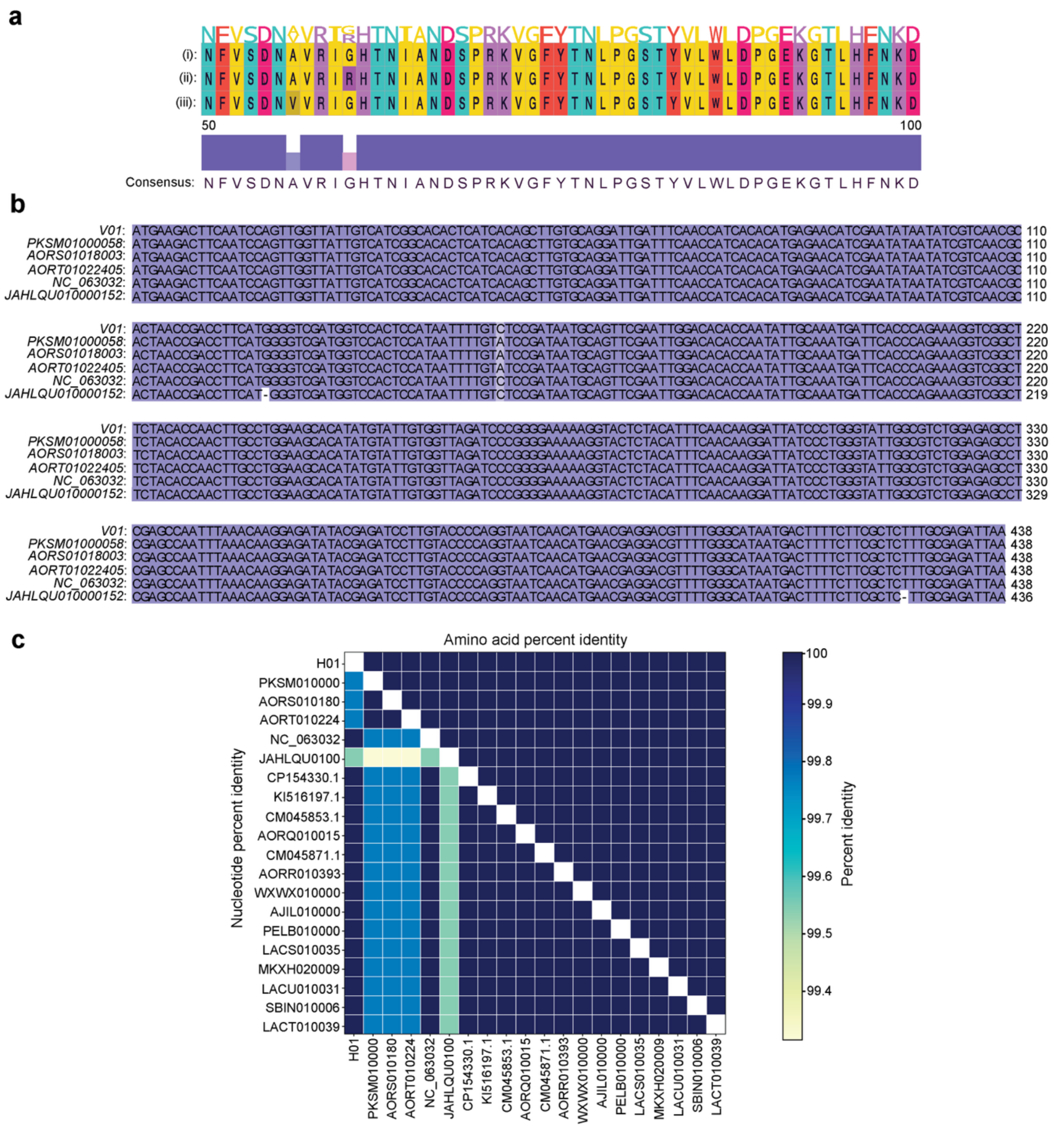

Supplementary Fig. 5. *PST130_P495001* is highly conserved in sequence across *Pst* isolates. a) Examination of the PST130_P495001 sequence across 273 *Pst* isolates identified three non-synonymous substitutions in 10 *Pst* isolates, representing two additional PST130_P495001 isoforms (ii and iii). Amino acids are coded in Clustalx colour format. **b)** Nucleotide sequence alignments of the *PST130_P495001* haplotype variants identified across 273 *Pst* isolates. All sequence alignments were constructed using MUSCLE via the EMBL-EBI Job Dispatcher website[3]. **c)** The *PST130_P495001* gene locus including 2 Kb upstream and downstream of the coding sequence and corresponding amino acid sequence is highly conserved across both haplotypes in all 19 *Pst* genome assemblies currently available (Supplementary Table **S4**).

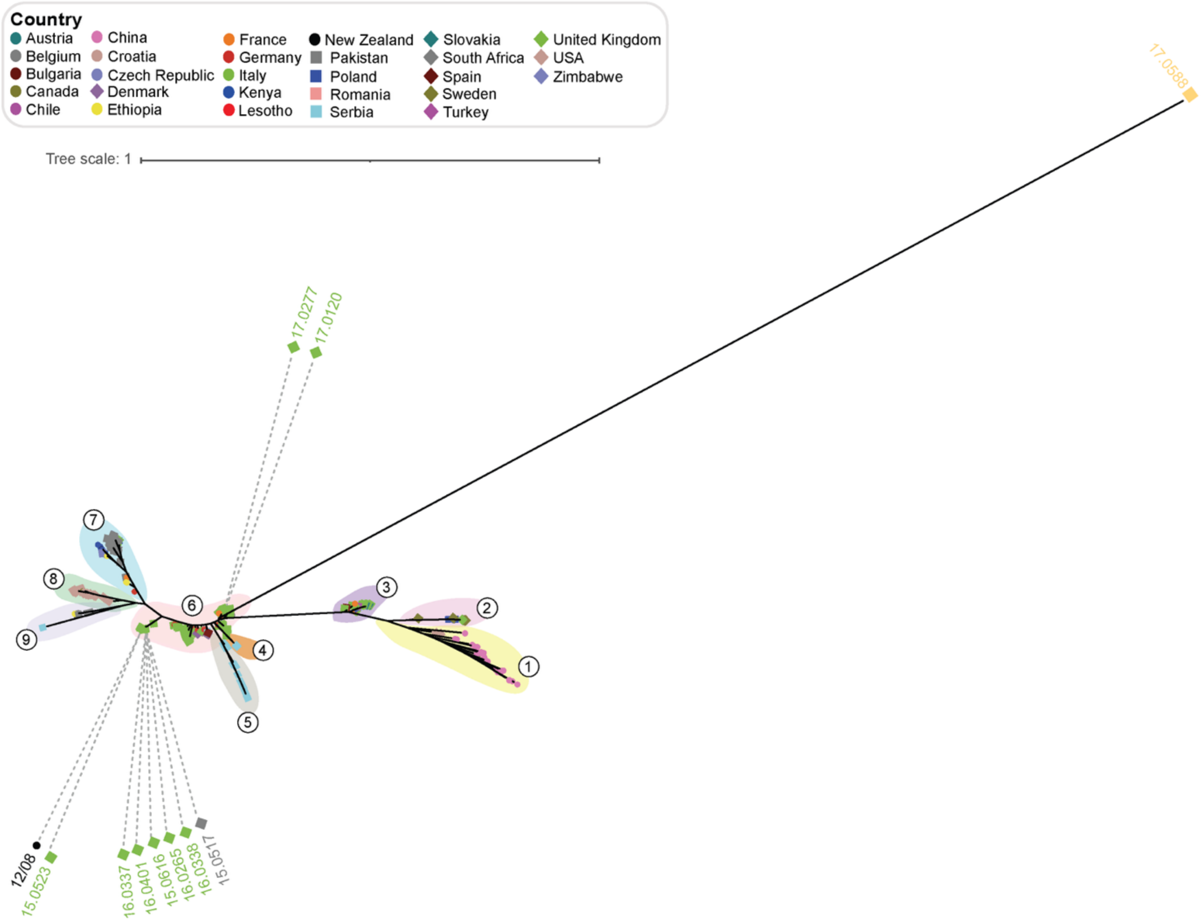

Supplementary Fig. 6. *Pst* isolates lacking *PST130_P495001* expression have arisen independently in the *PstS10* clade 6, including one *Pst* isolate from *Triticum durum*. Phylogenetic analysis of 438 global *Pst* isolates, including one collected on *T. durum* (17.0588), was performed using an approximate maximum-likelihood model. Scale bar indicates the mean number of nucleotide substitutions per site.

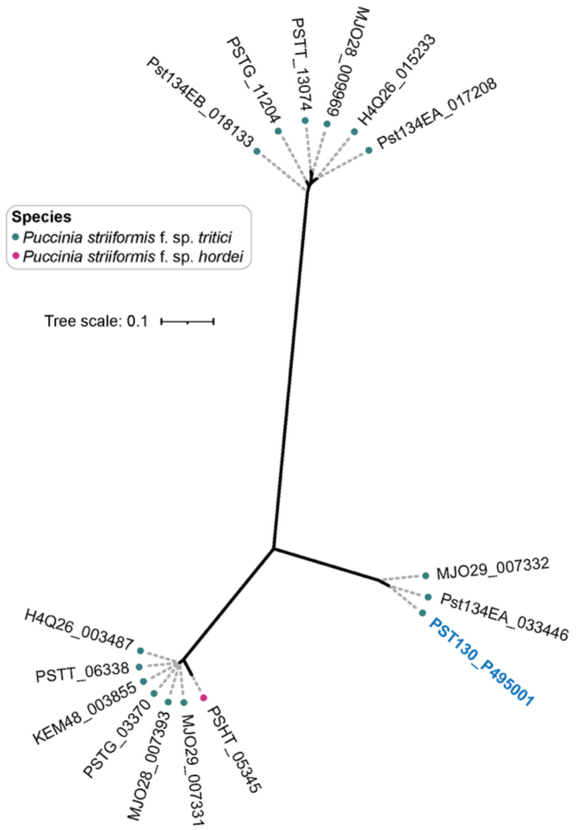

Supplementary Fig. 7. Proteins with sequence similarity to PST130_P495001 are limited to *Pst* and *Psh*. Phylogeny of the fifteen *Pst* and *Psh* proteins identified to share sequence similarity to PST130_P495001 in a BLAST sequence similarity search (e-value < 1e-5). The phylogenetic tree was generated with FastTree version 2.1.11. Scale bar indicates the mean number of nucleotide substitutions per site.

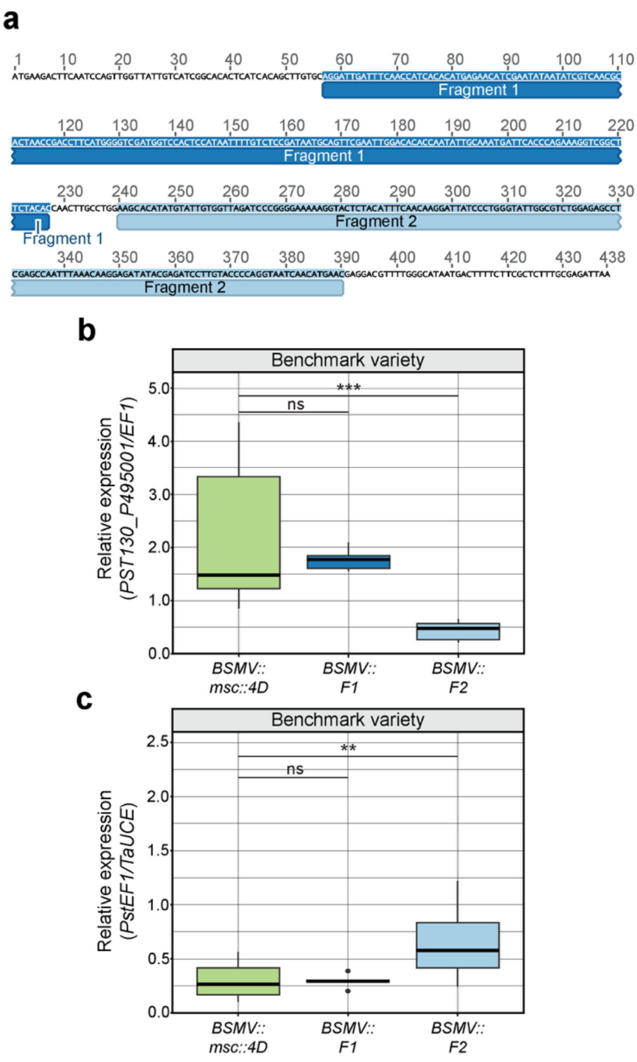

Supplementary Fig. 8. Host-induced gene silencing (HIGS) of *PST130_P495001*. a) Two fragments in two separate regions of the *PST130_P495001* gene (171-bp and 151-bp) were selected to induce silencing of *PST130_P495001*. **b)** Suppression of *PST130_P495001* expression was confirmed when using only fragment 2 (F2) of the *PST130_P495001* gene in a Benchmark-derived *Pst* isolate (DK229) during infection of the Benchmark variety. **c)** Suppression of *PST130_P495001* expression led to a minor increase in *Pst* (DK229_19) biomass during Benchmark infection. *PST130_P495001* silencing and fungal biomass was assessed using RT-qPCR at 4 days-post infection (dpi) and 11-15 days post-viral inoculation (dpvi) by comparing *PST130_P495001* to *PstEF1* expression (**b**) or *PstEF1* expression to the *TaUCE* wheat reference gene (**c**). Two to four samples each were evaluated for *BSMV::F1*, *BSMV::F2*, and *BSMV::msc4D*. Asterisks denote statistically significant differences between each pair of conditions (***: $p < 0.001$; **: $p < 0.01$; two-tailed *t*-test). Bar represents median value, box signifies the upper (Q3) and lower (Q1) quartiles, and whiskers are located at 1.5 times the interquartile range.

Supplementary Table 1. Virulence profiling of *Pst* isolates identified on Kalmar, Amboise and Benchmark wheat varieties. Three *Pst* isolates (DK267_17, FR167_20 and DK229_19) were used to inoculate the Kalmar, Amboise and Benchmark wheat varieties and a differential set of wheat lines, which harbour the following host yellow rust resistance genes: *Yr1, Yr2, Yr3, Yr4, Yr5, Yr6, Yr7, Yr8, Yr9, Yr10, Yr15, Yr17, Yr24, Yr25, Yr27, Yr32, YrSP, YrAmb* and *YrAvS*. Approximately 16 days post-inoculation (dpi), infection types were assessed. Figures and symbols indicate virulence corresponding to specific yellow rust resistance genes. –: Avirulence; Sd, Strubes Dickkopf; Su, Suwon-Omar; Sp, Spaldings Prolific; AvS, Avocet YrS; Amb, Ambition; Klm, Kalmar; Bmk, Benchmark and Abs, Amboise. ( ), indicates an intermediate interaction.

		**Virulence phenotyping**																					
***Pst* isolate**	**Race Group**	***1***	***2***	***3***	***4***	***5***	***6***	***7***	***8***	***9***	***10***	***15***	***17***	***24***	***25***	***27***	***32***	***Sd***	***Su***	***Sp***	***AvS***	***Amb***	***Klm***	***Bkm***	***Abs***
DK267_17	Kalmar	1	2	3	4	-	6	7	-	9	-	-	17	-	25	-	32	Sd	Su	Sp	AvS	-	Klm	-	(Abs)
DK229_19	Benchmark	1	2	3	4	-	6	7	-	9	-	-	17	-	25	-	32	Sd	Su	Sp	AvS	-	-	Bkm	Abs
FR167_20	Amboise	1	2	3	4	-	6	7	-	9	-	-	17	-	25	-	32	Sd	Su	Sp	AvS	-	Klm	Bkm	Abs

Supplementary Table 2. Assessment of PST130_P495001 for features typical of an effector protein. PST130_P495001 was examined for presence of a potential signal peptide (SP)[4] in the N-terminus and probability of being an apoplastic or cytoplasmic effector protein using machine learning[5].

Gene ID	SignalP prediction results[4]			EffectorP prediction results[5]	
	Signal Peptide (Sec/SPI)	Cleavage site	Probability	Cytoplasmic effector	Apoplastic effector
PST130_P495001	0.9997	Between 17 and 18 aa	0.965773	0.636	0.676

Supplementary Table 3. Details of the 19 *Pst* genome assemblies examined in this study.

Genome name	Isolate	Genome details	BioProject	Reference
Pst134E36_v1_pri	134E16A+17+33+	Chromosome level	PRJNA749614	[6]
ASM3951922v1	AZ2	Chromosome level	PRJNA1026770	[7]
ASM2516953v1	CYR34	Chromosome level	PRJNA807516	Unpublished
ASM2516955v1	93-210	Chromosome level	PRJNA807516	Unpublished
ASM1175075v1	DK09_11	Contig level	PRJNA595755	Unpublished
PucStrE137Aminus_diploid_v1.0	104 E137 A-	Contig level	PRJNA396589	Unpublished
ASM193660v2	38S102	Scaffold	PRJNA344021	Unpublished
ASM419432v1	11-281	Scaffold	PRJNA513913	Unpublished
ASM292020v1	93TX-2	Contig level	PRJNA422914	[8]
ASM200896v1	Race Yr9	Contig level	PRJNA277554	Unpublished
ASM200893v1	Race K	Contig level	PRJNA277553	Unpublished
ASM200892v1	Race 31	Contig level	PRJNA277552	Unpublished
ASM2586949v1	PST-130	Contig level	PRJNA650506	[8]
P_striiformis_V1	race PST-78	Scaffold	PRJNA41279	Unpublished
PST21_v01	PST21	Contig level	PRJNA181959	[9]
PST43_v01	PST43	Contig level	PRJNA181957	[10]
PST_08_21_v01	08/21	Contig level	PRJNA181960	[10]
PST_87_7_v01	87/7	Contig level	PRJNA181962	[10]
Cy321.0	CY32	Scaffold	PRJNA176877	Unpublished

Supplementary Table 4. Parentage of 20 wheat varieties that were infected with *Pst* isolates with regular or low *PST130_P495001* expression. The parental lineage of 20 *Pst*-infected wheat varieties was determined through analysis of an available wheat pedigree dataset[11].

Wheat variety	Male parent	Female parent	*PST130_P495001* expression levels
Robigus	1366	Z-836	Low
Zulu	CEBECO99080_x_Claire	Robigus	Low
Reflection	Denman	Oakley	Low
Myriad	NSL_00_0742	Nijinsky	Low
Invicta	NSL-WW-48	Robigus	Low
Britannia	Robigus	Cassius	Low
JB_Diego	STRU-2374	3351-B-2	Low
Claire	Wasp	Flame	Low
Scout	Z-435	Deben	Low
Oakley	Access	Aardvark(sib)_x_Robigus	Regular
KWS_Kielder	Brompton	Oakley	Regular
Vuka	CarstensVIII	Fanal_x_Graf-Toerring-II	Regular
Hereford	Deben	Solist	Regular
Kranich	Fanal	HeinesVII_x_Heines-2167-50	Regular
Cordiale	Malacca	Cadenza_x_Reaper	Regular
KWS_Santiago	Oakley	Sherborne	Regular
Solstice	Vivant	Rialto	Regular
Victo	W-1051-A	W-0010-E_x_79-W-810	Regular
Gallant	Xi-19	Malacca_x_Charger	Regular
Warrior	Robigus	CM-8228	Regular

Supplementary Table 5. Infection types following inoculation of 11 wheat varieties with *Pst* isolates collected on Kalmar or Benchmark. Two-week-old seedlings were infected with fresh *Pst* urediniospores from a Kalmar (DK267_17) and Benchmark (DK229_19) *Pst* isolate, and disease severity assessed 16 days post-inoculation (dpi).

		**Median infection type**	
**Wheat variety**	***PST130_P495001* expression**	***Pst Kalmar* isolate**	***Pst Benchmark* isolate**
Zulu	low	7.5 (S)	2.0 (R)
Hereford	low	7.0 (S)	2.0 (R)
JB Diego	low	6.8 (S)	2.0 (R)
Myriad	low	6.8 (S)	2.5 (R)
Scout	low	7.0 (S)	2.0 (R)
Reflection	low	6.8 (S)	2.5 (R)
Warrior	Regular	6.8 (S)	6.7 (S)
Cordiale	Regular	6.6 (S)	7.0 (S)
Solstice	Regular	7.0 (S)	6.5 (S)
RGT Reform	Regular	6.8 (S)	7.0 (S)
Alixan	Regular	7.0 (S)	7.0 (S)

Supplementary Table 6. Assessment of proteins from *Psh* and *Pgt* with structural homology to PST130_P495001 for features typical of an effector protein. Twelve proteins from *Puccinia graminis* f. sp. *tritici* and two from *Puccinia striiformis* f. sp. *hordei* with structural homology to PST130_P495001 were examined for the presence of a potential signal peptide (SP)[4] in the N-terminus and probability of being an apoplastic or cytoplasmic effector protein using machine learning[5].

		**SignalP prediction results[4]**			**EffectorP prediction results[5]**	
**Organism**	**Gene ID**	**Signal Peptide (Sec/SPI)**	**Cleavage site**	**CS Position**	**Cytoplasmic effector**	**Apoplastic effector**
Pst	A0A2S4VKV6	0.9757	Between 17 and 18 aa	0.9022	0.849	0.658
Psh	A0A2S4WAQ5	0.0000	-	-	0.767	0.723
Psh & *Pst*	A0A2S4WAX4	0.9997	Between 17 and 18 aa	0.9658	0.636	0.676
Pgt	A0A5B0LTC1	0.6459	Between 19 and 20 aa	0.3976	0.899	-
Pgt	A0A5B0LVR0	0.9995	Between 19 and 20 aa	0.9596	0.735	-
Pgt	A0A5B0LWJ1	0.9996	Between 19 and 20 aa	0.9769	0.815	0.592
Pgt	A0A5B0NWE5	0.9985	Between 19 and 20 aa	0.9315	0.869	0.721
Pgt	A0A5B0PBS3	0.9996	Between 19 and 20 aa	0.9730	0.778	0.607
Pgt	A0A5B0PDN7	0.9997	Between 19 and 20 aa	0.9670	0.664	-
Pgt	A0A5B0QFZ8	0.9994	Between 19 and 20 aa	0.9270	0.822	-
Pgt	A0A5B0RU29	0.9993	Between 19 and 20 aa	0.9293	0.894	-
Pgt	A0A5B0RX86	0.9996	Between 19 and 20 aa	0.9766	0.932	-
Pgt	A0A5B0SHF3	0.9996	Between 19 and 20 aa	0.9766	0.933	-
Pgt	E3JTQ7	0.9996	Between 19 and 20 aa	0.9766	0.932	-
Pgt	E3KSN7	0.9966	Between 19 and 20 aa	0.9135	0.979	-

Supplementary References

1 Schwessinger, B. *et al.* A Near-Complete Haplotype-Phased Genome of the Dikaryotic Wheat Stripe Rust Fungus *Puccinia striiformis* f. sp. *tritici* Reveals High Interhaplotype Diversity. *mBio* **9** (2018). https://doi.org:10.1128/mBio.02275-17

2 Bray, N. L., Pimentel, H., Melsted, P. & Pachter, L. Near-optimal probabilistic RNA-seq quantification. *Nat Biotechnol* **34**, 525-527 (2016). https://doi.org:10.1038/nbt.3519

3 Madeira, F. *et al.* The EMBL-EBI Job Dispatcher sequence analysis tools framework in 2024. *Nucleic Acids Research* **52**, W521-W525 (2024). https://doi.org:10.1093/nar/gkae241

4 Teufel, F. *et al.* SignalP 6.0 predicts all five types of signal peptides using protein language models. *Nat Biotechnol* **40**, 1023-+ (2022). https://doi.org:10.1038/s41587-021-01156-3

5 Sperschneider, J. & Dodds, P. N. EffectorP 3.0: Prediction of Apoplastic and Cytoplasmic Effectors in Fungi and Oomycetes. *Mol Plant Microbe Interact* **35**, 146-156 (2022). https://doi.org:10.1094/MPMI-08-21-0201-R

6 Schwessinger, B. *et al.* A Chromosome Scale Assembly of an Australian *Puccinia striiformis* f. sp. *tritici* Isolate of the *PstS1* Lineage. *Mol Plant Microbe In* **35**, 293-296 (2022). https://doi.org:10.1094/Mpmi-09-21-0236-A

7 Wang, J. R. *et al.* A fully haplotype-resolved and nearly gap-free genome assembly of wheat stripe rust fungus. *Sci Data* **11** (2024). https://doi.org:https://doi.org/10.1038/s41597-024-03361-6

8 Xia, C. J. *et al.* Genomic insights into host adaptation between the wheat stripe rust pathogen (*Puccinia striiformis* f. sp. *tritici*) and the barley stripe rust pathogen (*Puccinia striiformis* f. sp. *hordei*). *Bmc Genomics* **19** (2018). https://doi.org:https://doi.org/10.1186/s12864-018-5041-y

9 Vasquez-Gross, H., Kaur, S., Epstein, L. & Dubcovsky, J. A haplotype-phased genome of wheat stripe rust pathogen *Puccinia striiformis* f. sp. *tritici*, race PST-130 from the Western USA. *Plos One* **15** (2020). https://doi.org:https://doi.org/10.1038/s41597-024-03361-6

10 Cantu, D. *et al.* Genome analyses of the wheat yellow (stripe) rust pathogen *Puccinia striiformis* f. sp *tritici* reveal polymorphic and haustorial expressed secreted proteins as

candidate effectors. *Bmc Genomics* **14** (2013). https://doi.org:https://doi.org/10.1186/1471-2164-14-270

11 Fradgley, N. *et al.* A large-scale pedigree resource of wheat reveals evidence for adaptation and selection by breeders. *PLoS Biol* **17**, e3000071 (2019). https://doi.org:10.1371/journal.pbio.3000071
