# Supplementary material for: Virulence gains in the Puccinia striiformis f. sp. tritici PstS10 lineage correlate with expression polymorphism in a candidate Avr effector
Source: Commun Biol. 2026 Apr 13;9:790. doi: 10.1038/s42003-026-10018-0 (PMC13254256; doi:10.1038/s42003-026-10018-0)
Supplement: Supplementary file 2 — Description of Additional Supplementary Files [file 42003_2026_10018_MOESM2_ESM.pdf]

Description of Additional Supplementary File

File name: Supplementary data 1
Description: Description of Pst isolates analysed in this study.

File name: Supplementary data 2
Description: Percentage of reads aligned to the Pst reference genome assembly (isolate Pst104E137).

File name: Supplementary data 3
Description: Virulence profiling of Pst isolates identified on Kalmar, Amboise and Benchmark on the respective wheat varieties and the standard susceptible line Morocco. Infection types were assessed in replicate on a 0 – 9 scale, with 0-3 considered susceptible, 4-6 intermediate and 7-9 resistant.

File name: Supplementary data 4
Description: Virulence profiling of Pst isolates identified on Kalmar, Amboise and Benchmark wheat varieties. Infection types were assessed in replicate on a 0 – 9 scale, with 0-3 considered susceptible, 4-6 intermediate and 7-9 resistant.

File name: Supplementary data 5
Description: Pairwise comparisons of gene expression between Pst isolates from Amboise or Kalmar to those from Benchmark Pst isolates.

File name: Supplementary data 6
Description: Single nucleotide polymorphisms identified per base in PST130_P495001, Actin, Beta-tubulin and Elongation Factor 1 for 273 Pst isolates analysed.

File name: Supplementary data 7
Description: Metadata describing the 992 Pst RNA-seq datasets extracted from the Pst expression browser.

File name: Supplementary data 8
Description: PST130_P495001 expression across Pst-infected samples derived from 35 wheat varieties where at least four Pstinfected samples were available.

File name: Supplementary data 9
Description: Host-induced gene silencing of PST130_P495001 and fungal biomass assessed using RT-qPCR at 4 days-post infection (dpi) with Pst and 11-15 days post-viral inoculation (dpvi).

File name: Supplementary data 10
Description: Location of datasets used to generate figures.
