# Supplementary material for: Virulence gains in the Puccinia striiformis f. sp. tritici PstS10 lineage correlate with expression polymorphism in a candidate Avr effector
Source: Commun Biol. 2026 Apr 13;9:790. doi: 10.1038/s42003-026-10018-0 (PMC13254256; doi:10.1038/s42003-026-10018-0)
Supplement: Supplementary file 4 — Reporting summary [file 42003_2026_10018_MOESM4_ESM.pdf]

nature portfolio

Corresponding author(s): Diane G.O. Saunders

Last updated by author(s): 2026/03/14

Reporting Summary

Nature Portfolio wishes to improve the reproducibility of the work that we publish. This form provides structure for consistency and transparency in reporting. For further information on Nature Portfolio policies, see our Editorial Policies and the Editorial Policy Checklist.

Statistics

For all statistical analyses, confirm that the following items are present in the figure legend, table legend, main text, or Methods section.

n/a	Confirmed	
☐	☒	The exact sample size (*n*) for each experimental group/condition, given as a discrete number and unit of measurement
☐	☒	A statement on whether measurements were taken from distinct samples or whether the same sample was measured repeatedly
☐	☒	The statistical test(s) used AND whether they are one- or two-sided<br>*Only common tests should be described solely by name; describe more complex techniques in the Methods section.*
☒	☐	A description of all covariates tested
☒	☐	A description of any assumptions or corrections, such as tests of normality and adjustment for multiple comparisons
☐	☒	A full description of the statistical parameters including central tendency (e.g. means) or other basic estimates (e.g. regression coefficient) AND variation (e.g. standard deviation) or associated estimates of uncertainty (e.g. confidence intervals)
☐	☒	For null hypothesis testing, the test statistic (e.g. F, t, r) with confidence intervals, effect sizes, degrees of freedom and P value noted<br>*Give P values as exact values whenever suitable.*
☒	☐	For Bayesian analysis, information on the choice of priors and Markov chain Monte Carlo settings
☒	☐	For hierarchical and complex designs, identification of the appropriate level for tests and full reporting of outcomes
☒	☐	Estimates of effect sizes (e.g. Cohen's *d*, Pearson's *r*), indicating how they were calculated

Our web collection on statistics for biologists contains articles on many of the points above.

Software and code

Policy information about availability of computer code

Data collection	All custom computer code and the newick files for the phylogenies have been deposited on GitHub (https://github.com/SaundersLab/PST130_P495001-paper). All open source software including versions is included in the Materials and Methods section of the manuscript.
Data analysis	This is detailed extensively in the Materials and Methods section of the manuscript.

For manuscripts utilizing custom algorithms or software that are central to the research but not yet described in published literature, software must be made available to editors and reviewers. We strongly encourage code deposition in a community repository (e.g. GitHub). See the Nature Portfolio guidelines for submitting code & software for further information.

Data

Policy information about availability of data

All manuscripts must include a data availability statement. This statement should provide the following information, where applicable:

- Accession codes, unique identifiers, or web links for publicly available datasets
- A description of any restrictions on data availability
- For clinical datasets or third party data, please ensure that the statement adheres to our policy

The sequence data that supports the findings of this study has been deposited in the Sequence Read Archive (SRA: PRJNA1231252). All custom computer code and the newick files for the phylogenies have been deposited on GitHub (https://github.com/SaundersLab/PST130_P495001-paper)

Research involving human participants, their data, or biological material

Policy information about studies with human participants or human data. See also policy information about sex, gender (identity/presentation), and sexual orientation and race, ethnicity and racism.

Reporting on sex and gender	N/A
Reporting on race, ethnicity, or other socially relevant groupings	N/A
Population characteristics	N/A
Recruitment	N/A
Ethics oversight	N/A

Note that full information on the approval of the study protocol must also be provided in the manuscript.

Field-specific reporting

Please select the one below that is the best fit for your research. If you are not sure, read the appropriate sections before making your selection.

☒ Life sciences ☐ Behavioural & social sciences ☐ Ecological, evolutionary & environmental sciences

For a reference copy of the document with all sections, see nature.com/documents/nr-reporting-summary-flat.pdf

Life sciences study design

All studies must disclose on these points even when the disclosure is negative.

Sample size	The number of Pst isolates utilised that were derived from Kalmar, Amboise and Benchmark wheat varieties were determined by the number available.
Data exclusions	The only point of data exclusion in the study was during the assessment of PST130_P495001 expression in the 992 global Pst isolates, where Datasets with transcript counts below 15% of the median for these housekeeping genes were excluded from further analysis, resulting in 888 Pst datasets.
Replication	All replicates for each experiment are displayed in the manuscript.
Randomization	N/A
Blinding	Pathology scoring was conducted blinded.

Reporting for specific materials, systems and methods

We require information from authors about some types of materials, experimental systems and methods used in many studies. Here, indicate whether each material, system or method listed is relevant to your study. If you are not sure if a list item applies to your research, read the appropriate section before selecting a response.

Materials & experimental systems

n/a	Involved in the study	
☒	☐	Antibodies
☒	☐	Eukaryotic cell lines
☒	☐	Palaeontology and archaeology
☒	☐	Animals and other organisms
☒	☐	Clinical data
☒	☐	Dual use research of concern
☐	☒	Plants

Methods

n/a	Involved in the study	
☒	☐	ChIP-seq
☒	☐	Flow cytometry
☒	☐	MRI-based neuroimaging

Dual use research of concern

Policy information about dual use research of concern

Hazards

Could the accidental, deliberate or reckless misuse of agents or technologies generated in the work, or the application of information presented in the manuscript, pose a threat to:

No	Yes	
☒	☐	Public health
☒	☐	National security
☐	☒	Crops and/or livestock
☒	☐	Ecosystems
☒	☐	Any other significant area

Hazards: The introduction of the isolates of Puccinia striiformis f. sp. tritici (Pst) described into global regions where they are not currently prevalent could pose a risk of heightened disease outbreaks, depending on the susceptibility of the wheat crops in the region.

For examples of agents subject to oversight, see the United States Government Policy for Institutional Oversight of Life Sciences Dual Use Research of Concern.

Experiments of concern

Does the work involve any of these experiments of concern:

No	Yes	
☒	☐	Demonstrate how to render a vaccine ineffective
☒	☐	Confer resistance to therapeutically useful antibiotics or antiviral agents
☒	☐	Enhance the virulence of a pathogen or render a nonpathogen virulent
☒	☐	Increase transmissibility of a pathogen
☒	☐	Alter the host range of a pathogen
☒	☐	Enable evasion of diagnostic/detection modalities
☒	☐	Enable the weaponization of a biological agent or toxin
☒	☐	Any other potentially harmful combination of experiments and agents

Precautions and benefits

Biosecurity precautions: At JIC we hold an appropriate licence issued by DEFRA to import, hold, multiply etc worldwide isolates of the wheat yellow rust pathogen Puccinia striiformis f. sp. tritici (Pst) and Puccina graminis f. sp. tritici (Pgt), among others (licence number 51103/197332-9). All experiments followed the standard operating procedures developed in line with the licence conditions within the Saunders laboratory.

Biosecurity oversight: All procedures are regularly reviewed and inspected by DEFRA as part of the annual review of our licence.

Benefits: N/A

Communication benefits: N/A

Plants

Seed stocks: Kalmar, Amboise, and Benchmark wheat varieties were sourced from Nordic Seed, Denmark, Blackman Agricultural Ltd, UK and Sejet Plant Breeding, Denmark, respectively. All other wheat varieties were obtained from central collections held at the Global Rust Reference Centre (Denmark) or John Innes Centre (UK).

Novel plant genotypes: N/A

Authentication: N/A
